# The Network Pharmacology of Rosmarinic Acid and Elucidating the Wound Healing Potential of a Rosmarinic Acid-Enriched *Trachystemon orientalis* Extract

**DOI:** 10.3390/ph19101633

**Published:** 2026-10-15

**Authors:** Merve Badem, Şeyda Kanbolat, Nevin Ulas Çolak, Tuğba Subaş, Sıla Özlem Şener, Mohammed Ashkar, Ufuk Özgen

**Affiliations:** 1 Department of Biochemistry, Faculty of Pharmacy, Karadeniz Technical University, Trabzon 61080, Türkiye; seydaakkaya@ktu.edu.tr; 2 Department of Analytical Chemistry, Faculty of Pharmacy, Karadeniz Technical University, Trabzon 61080, Türkiye; nevinulas@ktu.edu.tr; 3 Department of Pharmacognosy, Faculty of Pharmacy, Karadeniz Technical University, Trabzon 61080, Türkiye; tugbasubas@ktu.edu.tr (T.S.); uozgen@ktu.edu.tr (U.Ö.); 4 Department of Pharmacognosy, Faculty of Pharmacy, University of Health Sciences, Ankara 06018, Türkiye; silaozlem.sener@sbu.edu.tr; 5 Department of Biochemistry, Institute of Health Sciences, Karadeniz Technical University, Trabzon 61080, Türkiye; mohammedashkar.0077@gmail.com

**Keywords:** *Trachystemon*, response surface methodology, scratch assay, rosmarinic acid

## Abstract

**Background/Objectives:** *Trachystemon orientalis* (L.) D. Don has been reported to be used in folk medicine for the treatment of inflammatory wounds and for its contained rosmarinic acid, a phenolic compound known for its antioxidant, anti-inflammatory, antimicrobial, and wound healing properties. This study aimed to obtain a rosmarinic acid-enriched extract from the aerial parts of *T. orientalis* and evaluate its in vitro wound healing potential. **Methods:** Extraction conditions were optimized using fractional factorial design and Box–Behnken optimization. Rosmarinic acid content was quantified by RP-HPLC. A rosmarinic acid-enriched extract was compared with a maceration extract and isolated rosmarinic acid. Elastase, hyaluronidase, and collagenase inhibitory activities were determined. Cytotoxicity was evaluated on L929 fibroblasts using the MTT assay, and non-cytotoxic concentrations were assessed for their effects on in vitro wound healing by scratch assay. In addition, in silico target prediction and network pharmacology analyses were performed using rosmarinic acid. **Results:** Optimization successfully yielded a rosmarinic acid-enriched extract from *T. orientalis*. The rosmarinic acid-enriched extract inhibited wound-related enzymes and enhanced wound closure in the scratch assay. Network pharmacology analysis showed putative molecular targets and signaling pathways potentially associated with the wound healing activity of rosmarinic acid, especially those involved in inflammatory responses, extracellular matrix remodeling, and tissue regeneration. **Conclusions:** The rosmarinic acid-enriched extract exhibited promising in vitro wound healing-related activity through the inhibition of extracellular matrix degrading enzymes and promotion of wound closure. Furthermore, network pharmacology analysis provided preliminary mechanistic insights based on computational predictions.

## 1. Introduction

A wound is defined as the disruption of the anatomical integrity and functional structure of tissue caused by burns, cuts, penetrating injuries, or various biochemical and physiological factors. Wound healing is a complex process involving successive and interacting stages: hemostasis, inflammation, proliferation, and remodeling [1,2]. The main factors that influence the wound healing process include oxygenation, infection, blood flow to the wound area, age, stress, genetic and immunological conditions, diabetes, nutritional status, and the use of certain medications. In addition, wound healing is regulated by a complex signaling network involving cytokines such as TNF-α and interleukins as well as growth factors including platelet-derived growth factor (PDGF), fibroblast growth factor (FGF), transforming growth factor-beta (TGF-*β*), and epidermal growth factor (EGF). These cytokines and growth factors stimulate the migration of inflammatory cells and fibroblasts to the wound area, activate angiogenesis, and contribute to the formation of the extracellular matrix (ECM). Furthermore, matrix metalloproteinases (MMPs) regulate the degradation and remodeling of the ECM, thereby playing a crucial role in re-epithelialization and tissue repair. In conclusion, the successful progression of wound healing depends on the coordinated interaction and balance among cellular components, growth factors, and enzymatic processes [3]. Wound healing processes can be evaluated by various in vitro and in vivo methods. Among in vitro approaches, fibroblast and keratinocyte cell culture tests are among the most frequently preferred models for studying wound healing mechanisms [4]. The scratch (wound healing) assay, applied in single-layer cell cultures, is commonly employed to evaluate the migration and proliferation of epithelial cells. This method provides valuable insights into wound healing by measuring the rate at which cells migrate and proliferate to close the artificially created scratch [5]. In addition, evaluating the inhibitory effects on enzymes such as hyaluronidase, collagenase, and elastase—responsible for the enzymatic degradation of ECM components, including hyaluronic acid, collagen, and elastin—represents another frequently used in vitro method in wound healing studies [6].

Experimental design plays an important role in ensuring that processes are conducted more efficiently and reliably. Identifying the parameters affecting a system and determining their effects enhances the value of the obtained results. In conventional approaches, examining parameters individually often leads to the neglect of interactions between them. In contrast, experimental design methods enable the evaluation of interactions among factors and their combined effects on the response. Among optimization techniques, response surface methodology (RSM) is frequently preferred, particularly in green chemistry applications, as it allows the simultaneous evaluation of multiple parameters with a minimal number of experiments [7].

*Trachystemon orientalis* (L.) D. Don, which is widely distributed in Türkiye, particularly in the Black Sea Region, is known by various local names such as “kaldırak” and “hodan” [8]. It has been reported that the leaves and stems of the species are non-toxic and edible, and in the region, its flowers, budding stems, and leaves are consumed as food [9,10]. Additionally, it has been demonstrated that the species is used as a folk remedy for various ailments, namely depression, rheumatism, and inflammation, due to its diuretic, expectorant, and sudorific effects; furthermore, a decoction prepared from the species’ roots in combination with other medicinal plants is administered internally for breast cancer and stomach disorders [10,11,12,13]. It has been suggested that the species possesses various biological activities, including allelopathic, antiviral, antimicrobial, antidiabetic, antihyperlipidemic, and antimutagenic effects [14,15,16,17]. The species, traditionally used for digestive problems, has been reported to exhibit inhibitory effects on digestive enzymes such as *α*-amylase, *α*-glucosidase, pancreatic lipase, and cholesterol esterase, and its rhizomes have been reported to possess anticancer activity [16,17,18]. Additionally, the plant’s use in the treatment of inflammatory wounds has been noted in an ethnobotanical study [13].

Phytochemical studies indicate that *T. orientalis* is rich in phenolic compounds, flavonoids, and anthocyanins and exhibits strong antioxidant activity; therefore, it can be evaluated as a nutritional supplement and as a natural antioxidant source in the food and cosmetic industries [19,20]. It has also been reported to have a high mineral and protein content [21].

Several previous phytochemical studies have consistently demonstrated that rosmarinic acid (RA) is one of the predominant phenolic compounds in both the aerial and underground parts of *T. orientalis* and is considered one of the major contributors to the biological activities of the species [16,17,22]. In the RP-HPLC analysis conducted by Bıyık et al., RA was identified as the major compound in methanolic extracts of the aerial parts and roots, while more recently isolation studies conducted by Koz et al. and Subaş et al. successfully isolated RA from the underground and aerial parts, respectively, providing additional phytochemical evidence for its prominent occurrence in the species [16,17,22]. Therefore, *T. orientalis* has consistently been recognized in the literature as a species characterized by a high occurrence of RA.

Studies in the literature have shown that RA supports wound healing; it can be more effective than dexpanthenol in reducing wound size in topical applications and positively affects the inflammation, proliferation, and remodeling phases [23,24]. In addition, the antioxidant, anti-inflammatory, and antimicrobial properties of RA play an important role in reducing oxidative damage and supporting tissue repair in the wound healing process [25,26,27,28].

Considering its high RA content and the reported ethnomedicinal uses, *T. orientalis* appears to be a promising candidate for wound healing applications. However, to the best of our knowledge, no studies have directly investigated the wound healing potential of the species. Therefore, the objective of the present study was to investigate the in vitro wound healing potential of a rosmarinic acid-enriched extract (TO-O) obtained from *T. orientalis*. For comparative purposes, the conventional maceration extract (TO-M) prepared from the aerial parts of *T. orientalis* and isolated RA were also evaluated under the same experimental conditions. In addition, the present study, a network pharmacology approach, including protein–protein interaction (PPI) network construction, hub gene identification, functional enrichment analysis, and drug–target–pathway–disease network modeling, was employed to explore the potential molecular mechanisms associated with the wound healing activity of RA.

## 2. Results and Discussion

### 2.1. Extraction Optimization and Model Analysis

**Figure 1:**
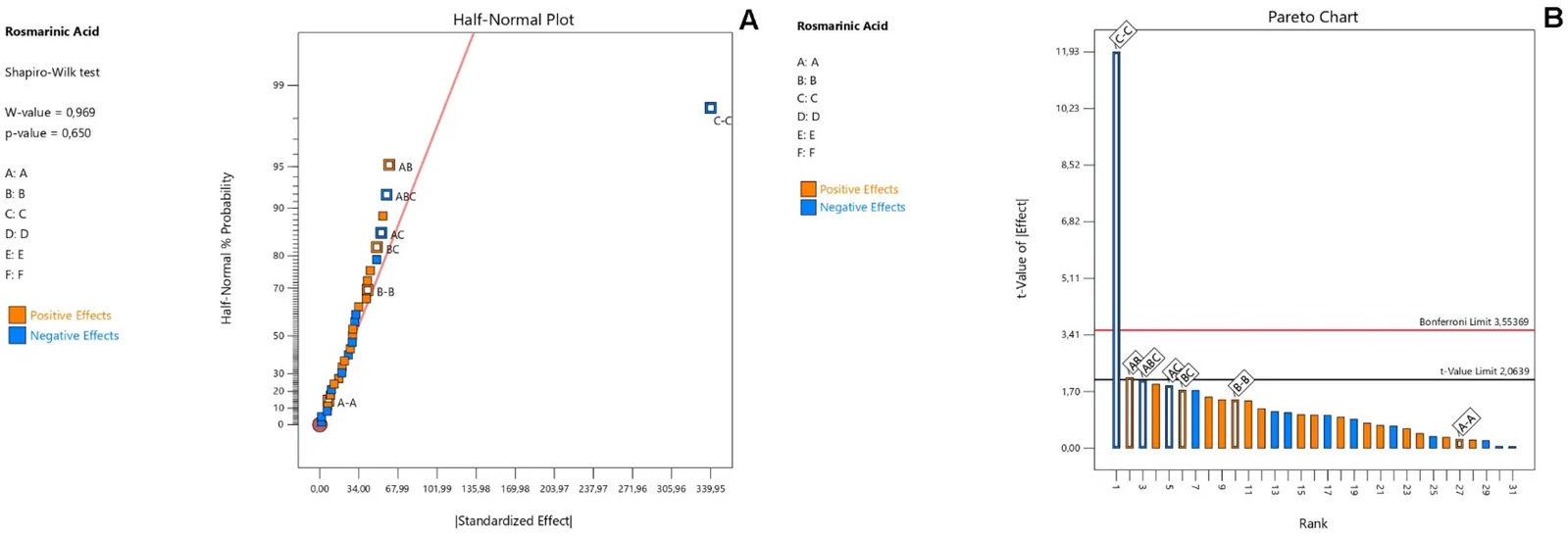
Shapiro–Wilk test plot (**A**) and Pareto chart of factors (**B**).

To determine the effective parameters in the extraction process of RA from the aerial parts of the species, a two-level, six-factor fractional factorial experimental design was applied, and a total of 32 randomized experiments were conducted. The obtained experimental data were analyzed using Design Expert 11.1.2. Percentage area values of RA were considered as response variables. The effect levels and significance levels of the parameters are presented in Figure 1 and Table 1. Among the six evaluated parameters, extraction time, temperature, and solvent volume were found to have the most pronounced effects on the response. The model was statistically significant (F = 22.84, *p* < 0.0001), indicating that the variability in the response was not due to random error. Analysis of variance (ANOVA) showed that terms C and AB were significant (*p* < 0.05), while non-significant terms (*p* > 0.10) were excluded to improve model simplicity without compromising hierarchy.

In Box–Behnken optimization, a total of 17 experiments were conducted in random order for three parameters with five replicates at the central point, and multiple regression analysis was applied to the data. ANOVA was used to statistically evaluate the model. The reduced ANOVA squared model data are shown in Table 2.

For the Box–Behnken design, a quadratic model provided the best fit to the experimental data (Table 2). The model was highly significant (F = 37.82, *p* < 0.0001), with B, C, and C^2^ identified as significant terms (*p* < 0.05), indicating the contribution of both linear and quadratic effects. The lack-of-fit test was not significant (F = 1.18, *p* > 0.05), confirming that the model adequately represents the experimental data within the studied range.

**Figure 2:**
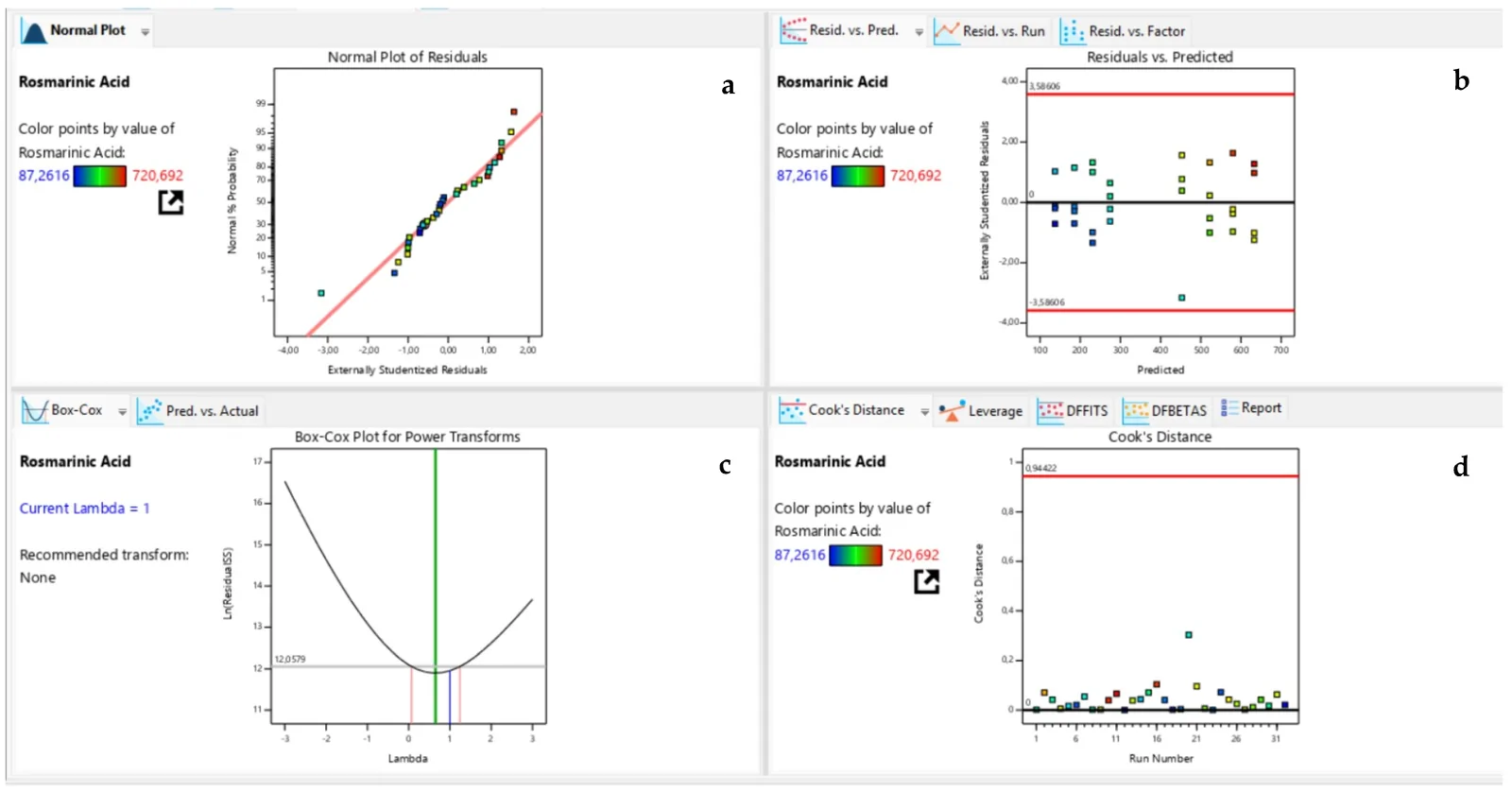
Model diagnostic plots for the Box–Behnken design: (**a**) normal plot of residuals, (**b**) residuals vs. predicted, (**c**) Box–Cox plot for power transforms, (**d**) Cook’s distance.

The adequacy of the model was further supported by diagnostic analyses (Figure 2). The normal probability plot of residuals showed a linear distribution, indicating that the normality assumption was satisfied. The residuals were randomly distributed around zero, confirming the absence of systematic error and the presence of constant variance. The Box–Cox plot (λ ≈ 1) suggested that no transformation of the response was required. In addition, Cook’s distance values indicated that no influential data points significantly affected the model.

As shown in Table 3, the quadratic model was selected based on the model selection criteria provided by the statistical software. The quadratic model showed a significant sequential *p*-value (*p* = 0.0065) and a non-significant lack-of-fit (*p* = 0.4234), indicating an adequate fit to the experimental data. In addition, it provided high adjusted and predicted R^2^ values (0.9539 and 0.8322, respectively). In contrast, the 2FI model did not provide a significant improvement (*p* = 0.5160), while the cubic model was aliased. Therefore, the quadratic model was recommended and selected for further analysis.

**Figure 3:**
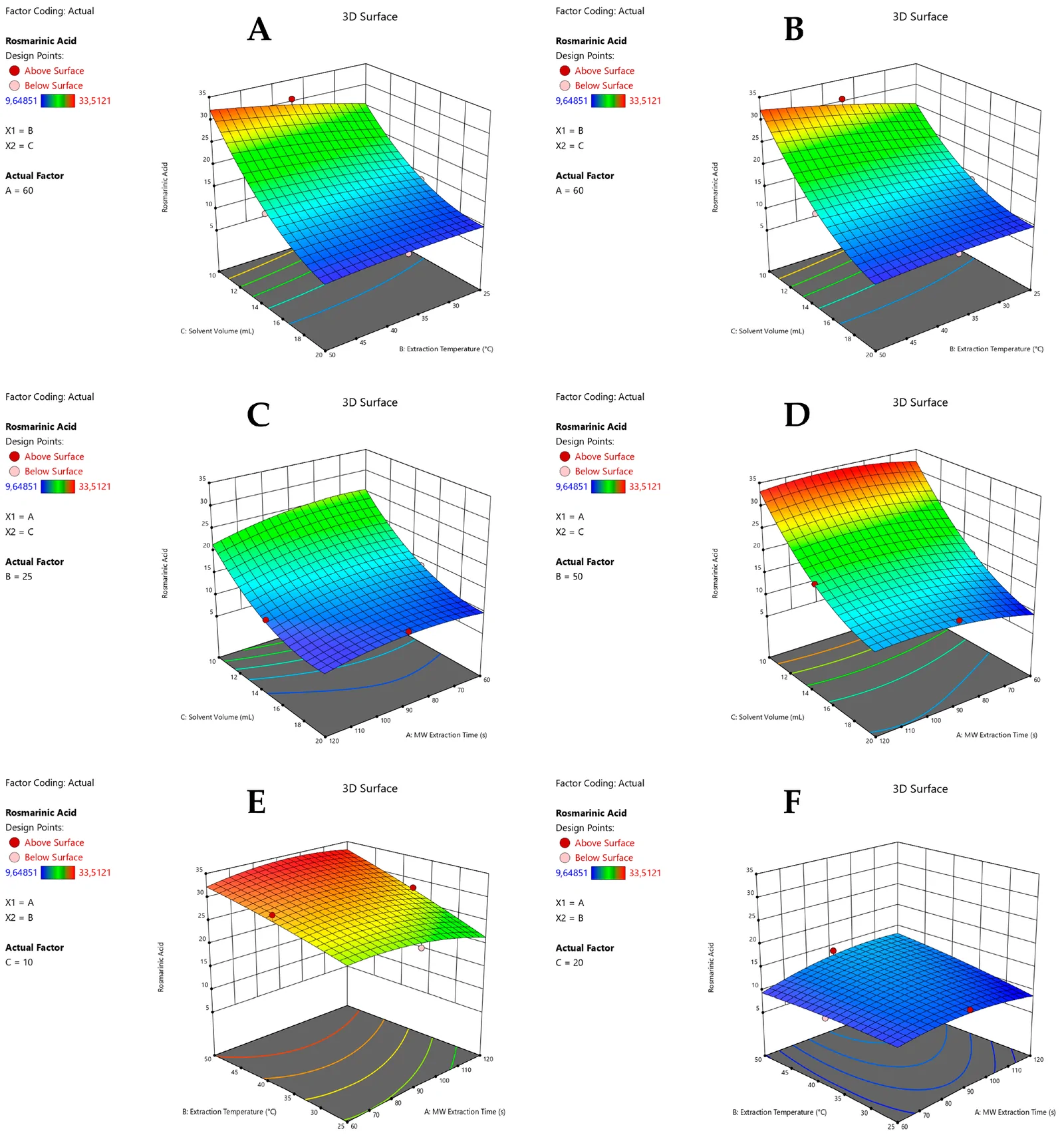
Model diagrams at the minimum and maximum levels of MW time (**A**,**B**), temperature (**C**,**D**), and solvent amount (**E**,**F**).

As shown in Figure 3, the interaction between extraction temperature, solvent volume, and extraction time significantly influenced RA yield. Response surface analysis revealed that increasing temperature enhanced extraction efficiency, likely due to improved solubility and mass transfer; however, excessively high temperatures may lead to the degradation of RA. Similarly, higher microwave power accelerated extraction, although prolonged exposure may increase the risk of thermal degradation. In contrast, solvent volume showed a negative effect on extraction yield. This behavior can be attributed to dilution effects, reduced concentration gradients, and decreased mass transfer efficiency at higher solvent volumes. Moreover, interactions between process variables may have contributed to this effect. Overall, the results demonstrate that extraction efficiency is governed by both linear and quadratic effects of the process variables. The developed model is statistically robust and provides a reliable tool for predicting and optimizing extraction conditions. Careful control of key parameters is therefore essential to achieve maximum yield while maintaining compound stability.

Factorial designs are commonly used as preliminary screening tools before response surface methodology to eliminate non-influential variables and identify the factors that most strongly affect phenolic compound extraction. Previous studies on RA extraction have reported drying temperature and storage duration as influential parameters, whereas extraction time showed no significant effect. In contrast, the present study identified both extraction time and temperature as important factors. This difference may be attributed to the extraction techniques employed. Previous studies used conventional maceration, whereas microwave-assisted extraction was applied in the present study. Because maceration may approach equilibrium during prolonged extraction, the influence of extraction time may become less pronounced than that observed in the shorter and more energy-intensive microwave-assisted process [29,30,31].

### 2.2. Targeted RP-HPLC Analysis of the Rosmarinic Acid-Enriched Extract

**Figure 4:**
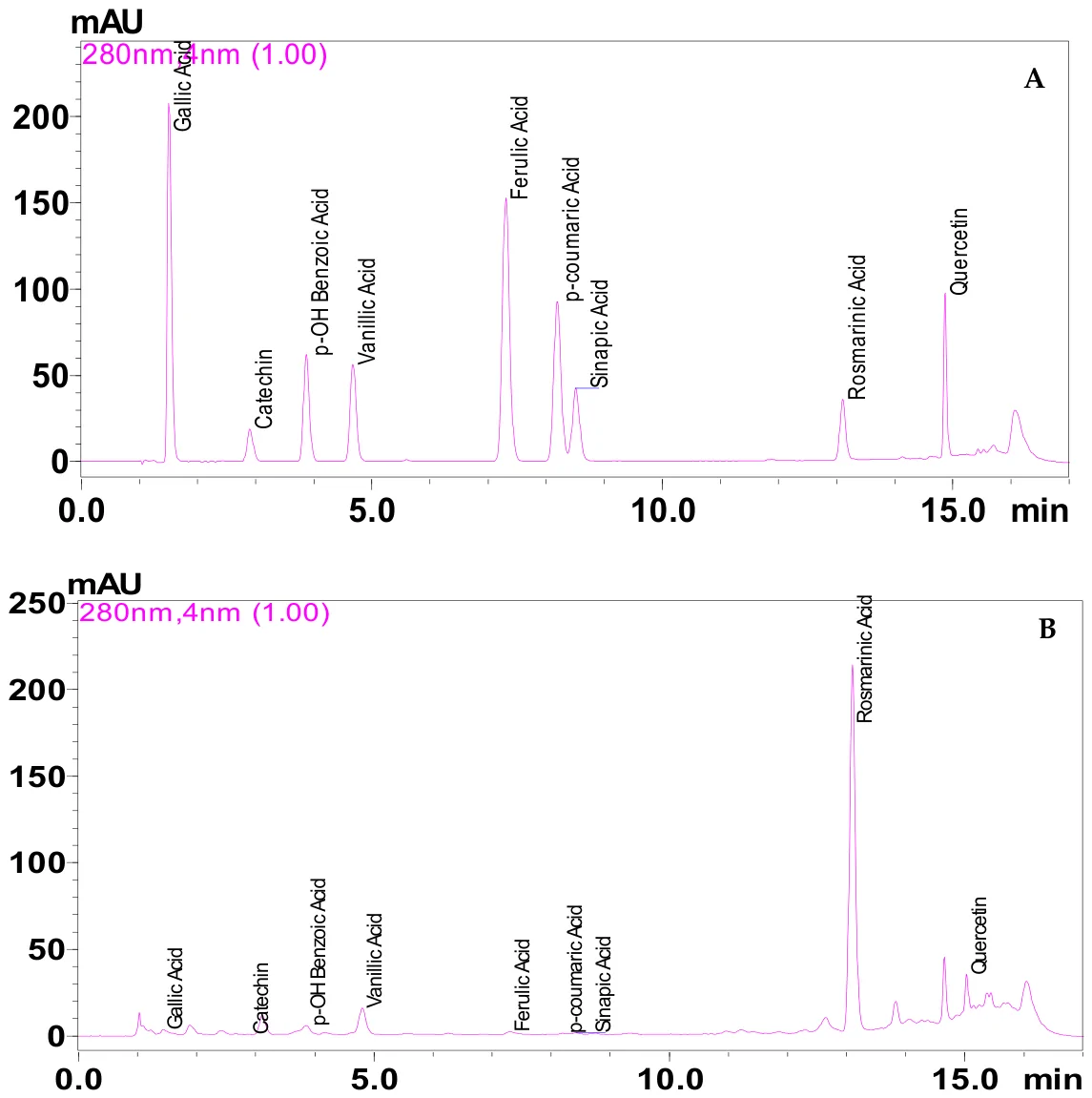
Chromatograms of the standard solution mixture (**A**) and TO-O (**B**).

The reliability of the RP-HPLC method used for quantitative analysis was confirmed by method validation performed according to the ICH Q2 (R2) guideline. Detailed validation results are presented in Supplementary File S1. The selected phenolic compounds of TO-O were determined as RA (550.10 mg/g dry extract), catechin (50.23 mg/g dry extract), vanillic acid (42.64 mg/g dry extract), *p*-hydroxybenzoic acid (15.10 mg/g dry extract), gallic acid (12.50 mg/g dry extract), quercetin (3.45 mg/g dry extract), ferulic acid (2.65 mg/g dry extract), sinapic acid (1.43 mg/g dry extract), and *p*-coumaric acid (1.38 mg/g dry extract). RA was the predominant quantified compound among the selected phenolic compounds analyzed of TO-O (Figure 4). The representative RP-HPLC chromatogram also revealed additional chromatographic peaks, indicating the presence of constituents other than the selected phenolic compounds analyzed in the present study. Further comprehensive phytochemical characterization will be required to identify these constituents.

### 2.3. Enzyme İnhibitory Effects of Extracts and Rosmarinic Acid

Elastase, hyaluronidase, and collagenase are among the major metalloproteinases responsible for the enzymatic degradation of ECM proteins. These enzymes play a critical role in maintaining the structural integrity, remodeling, and repair processes of tissues, as well as in the balance of ECM degradation and synthesis. In particular, in studies aimed at elucidating wound healing mechanisms, evaluating the inhibitory effects of these enzymes is a commonly preferred approach [2].

In this study, the inhibitory effects of TO-O obtained experimentally using an experimental design method, TO-M from the aerial parts of *T. orientalis*, and isolated RA on these enzymes were investigated. The IC_50_ values for enzyme inhibition of the samples are presented in Table 4.

The IC_50_ results showed that TO-O exhibited the strongest collagenase inhibition among the tested samples, with an IC_50_ value of 35.166 ± 7.713 µg/mL. Its activity was significantly higher than that of TO-M (66.863 ± 1.653 µg/mL) and was comparable to those of RA and the positive control. For elastase inhibition, RA and the positive control showed the strongest activities, followed by TO-O and TO-M. TO-O exhibited significantly stronger elastase inhibition than TO-M, with IC_50_ values of 96.846 ± 5.736 and 145.030 ± 8.681 µg/mL, respectively. RA showed the strongest hyaluronidase inhibition, whereas TO-O exhibited an inhibitory activity comparable to those of TO-M and the positive control. Overall, TO-O showed stronger collagenase and elastase inhibitory activities than the conventional maceration extract.

Several previous studies have demonstrated that extracts containing RA exhibit antioxidant, anti-inflammatory, and ECM protective properties. These extracts have consistently shown strong inhibitory activities against collagenase, elastase, and hyaluronidase, and antioxidant capacity [32,33,34]. In addition, these extracts have been reported to suppress inflammatory mediators, including NF-*κ*B and IL-6; increase fibroblast viability; protect cells against oxidative stress; and improve skin-related biological functions [33,34]. RA has been reported in various studies as an important compound in the protection of the ECM integrity by inhibiting collagenase, elastase, and hyaluronidase activities, promoting type I collagen synthesis, and suppressing matrix metalloproteinases (MMP-1, MMP-2, and MMP-9) [34,35,36,37].

### 2.4. In Vitro Cytotoxic and Wound Healing Effects of Extracts and Rosmarinic Acid

Wound healing, resulting from the disruption of tissue integrity, is a highly complex and multi-stage process. In this process, fibroblasts play an active role in stages ranging from the early inflammatory phase to ECM synthesis, making them critically important in tissue repair and the restoration of structural integrity. In particular, fibroblast migration and proliferation during the proliferative phase supports wound contraction and re-epithelialization processes, constituting one of the most important steps in wound closure. Therefore, fibroblast cells are among the commonly preferred cell lines for evaluating in vitro wound closure and tissue repair process [38].

**Figure 5:**
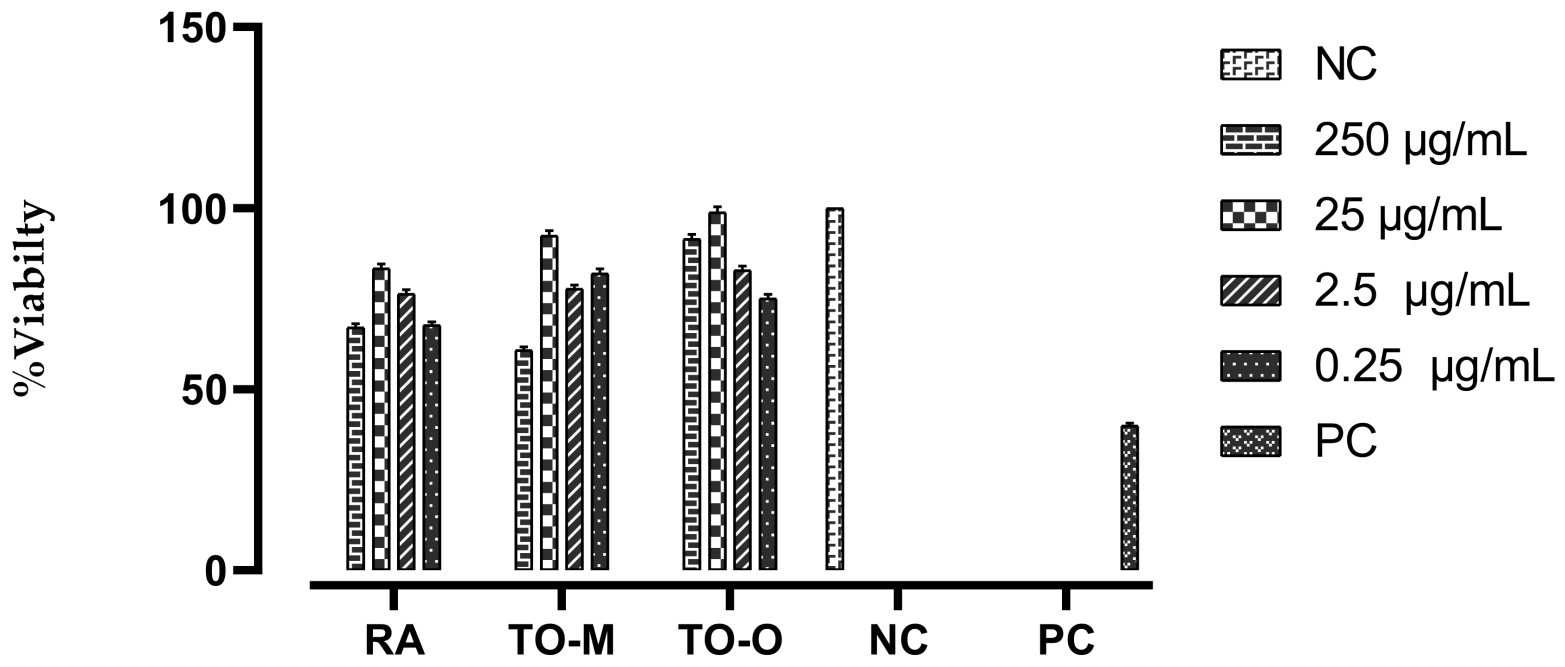
The results of cytotoxicity studies on L929 cells after 24 h of exposure. TO-O: rosmarinic acid-enriched extract of *T. orientalis*; TO-M: maceration extract of *T. orientalis*; RA: rosmarinic acid; PC: sodium lauryl sulfate; NC: 0.5% (*v*/*v*) methanol.

**Figure 6:**
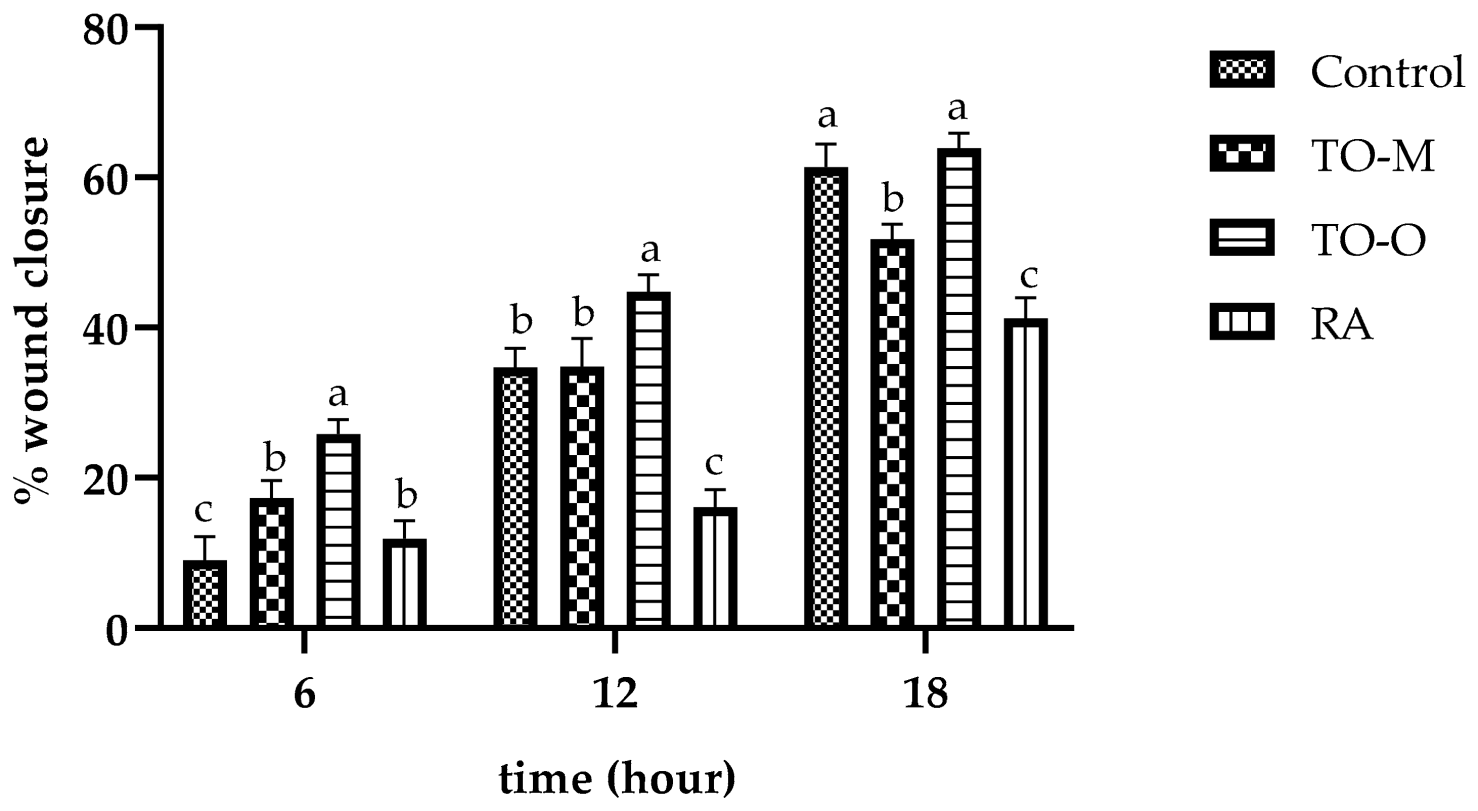
A comparison of the time-dependent % closure rates of samples. Values bearing different lowercase letters within the same column are significantly different according to Tukey’s multiple-comparison test (*p* < 0.0001), whereas values bearing the same letter are not significantly different. TO-O: Rosmarinic acid-enriched extract of *T. orientalis*; TO-M: maceration extract of *T. orientalis*; RA: rosmarinic acid; Control: cells maintained in culture medium without exposure to any test sample.

**Figure 7:**
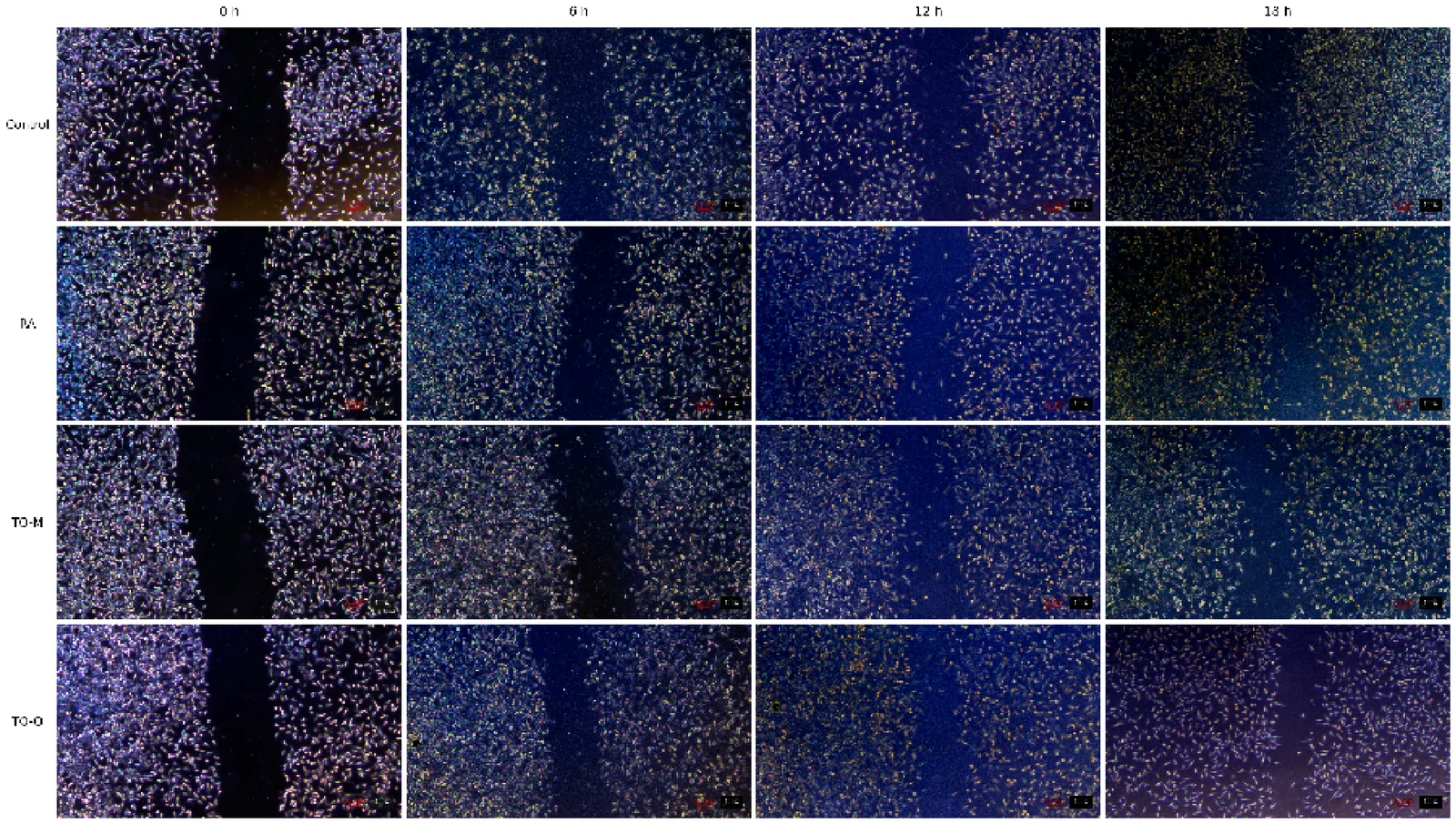
Time-dependent wound healing activity of samples after 0, 6, 12 and 18 h of incubation (4×). TO-O: Rosmarinic acid-enriched extract of *T. orientalis*; TO-M: maceration extract of *T. orientalis*; RA: rosmarinic acid; Control: cells maintained in culture medium without exposure to any test sample. Scale bars represent 100 µm (0.1 mm) in all images.

In the present studies, the cytotoxic effects of TO-O obtained through the optimized extraction process, TO-M from the aerial parts of *T. orientalis*, and isolated RA were evaluated using the MTT test; their potential effects on wound closure were subsequently assessed using the scratch assay on the L929 fibroblasts. According to the MTT test results, cell viability was determined to be above 50% at all tested concentrations in all tested samples. In particular, at a concentration of 25 µg/mL, cell viability values were observed to be quite close to the negative control group. These findings indicate that the samples examined did not show a significant cytotoxic effect within the concentration range evaluated (Figure 5). In the scratch assay, the wound closure effects of the samples were investigated using the concentrations at which cell viability was determined to be highest in the MTT test. The wound area was photographed at 0, 6, 12, and 18 h and wound closure percentages were calculated using ImageJ (version 1.54g; National Institutes of Health, Bethesda, MD, USA) based on decreasing the scratched area. As seen in Table 5 and Figure 6 and Figure 7, TO-O generally exhibited higher wound closure percentages than TO-M and isolated RA throughout the experimental period.

Since a proliferation inhibitor was not included in the present scratch assay, the wound closure may state the combined effects of fibroblast migration and proliferation. For this reason, the observed effects were interpreted as improved wound closure rather than migration alone. Though RA was selected as the marker compound based on previous phytochemical studies consistently identifying it as one of the predominant compounds of *T. orientalis* and on its well documented wound healing properties, the activity observed in the scratch assay cannot be attributed simply to RA. Other characterized and uncharacterized compounds present in the extract may also contribute to the observed effects through additive or synergistic interactions. However, the relative contribution of these constituents remains to be elucidated in future studies.

### 2.5. In Silico Target Prediction and Network Pharmacology Analysis of Rosmarinic Acid

Since the extraction process was optimized based on RA content, and previous phytochemical studies consistently identified rosmarinic acid as a predominant phenolic constituent of *T. orientalis*, this compound was selected for network pharmacology analysis. The in silico analysis was performed to complement the experimental findings by exploring the potential molecular targets and signaling pathways specifically associated with RA. It was not intended to represent the pharmacological effects of the whole extract or to establish experimentally validated molecular mechanisms.

#### 2.5.1. ADME/Toxicity Profile of Rosmarinic Acid

The ADME/toxicity profile of RA was predicted using SwissADME (https://www.swissadme.ch/; accessed on 6 April 2026) and ProTox-3.0. The compound has a molecular weight of 360.31 g/mol, which complies with the Lipinski molecular weight criterion. It contains 26 heavy atoms, including 12 aromatic heavy atoms, and possesses seven rotatable bonds, indicating moderate structural flexibility. The consensus logP of 1.58 suggests balanced lipophilicity. However, the TPSA value of 144.52 Å^2^ indicates high polarity, consistent with the predicted low gastrointestinal (GI) absorption. The SwissADME-predicted low gastrointestinal absorption of RA is consistent with pharmacokinetic reports in the literature. Oral absolute bioavailability of RA has been reported as low as 1–1.7% in rats, reflecting rapid Phase I hydrolysis and extensive Phase II conjugation (glucuronidation, sulfation, and methylation), while the unabsorbed fraction is further metabolized by colonic microbiota into caffeic acid and related phenolic metabolites [39]. These pharmacokinetic limitations, common among phenylpropanoid and caffeic-acid-derived compounds due to their hydroxyl- and carboxyl-rich structures, represent a major translational barrier for their therapeutic application [40]. Emerging strategies such as esterification and nanoscale co-assembly of natural phenolic compounds into non-covalent nanocomplexes have shown promise in enhancing their oral absorption and systemic exposure [40], and such approaches, together with the broader evidence-based translational framework being established for food-medicine homologous substances [41], may represent valuable directions for improving the oral bioavailability and therapeutic applicability of RA in future wound healing formulations.

RA is soluble in water and is not predicted to be a substrate of P-glycoprotein (P-gp), which may reduce the risk of active efflux as shown in Supplementary File S2 (Table S2). Toxicity predictions indicated that the compound is non-mutagenic (*p* = 0.85), non-carcinogenic (*p* = 0.66), non-cytotoxic (*p* = 0.90), and non-ecotoxic (*p* = 0.79). Nevertheless, potential concerns were predicted for immunotoxicity (active, *p* = 0.93) and BBB permeability (active, *p* = 0.62), while clinical toxicity showed a borderline prediction (*p* = 0.59). These results suggest that RA exhibits a generally acceptable in silico safety profile, although further experimental studies are required to clarify its immunological and systemic safety (Supplementary File S2, Table S3 and Figure S1).

#### 2.5.2. Common Targets Prediction of Rosmarinic Acid

**Figure 8:**
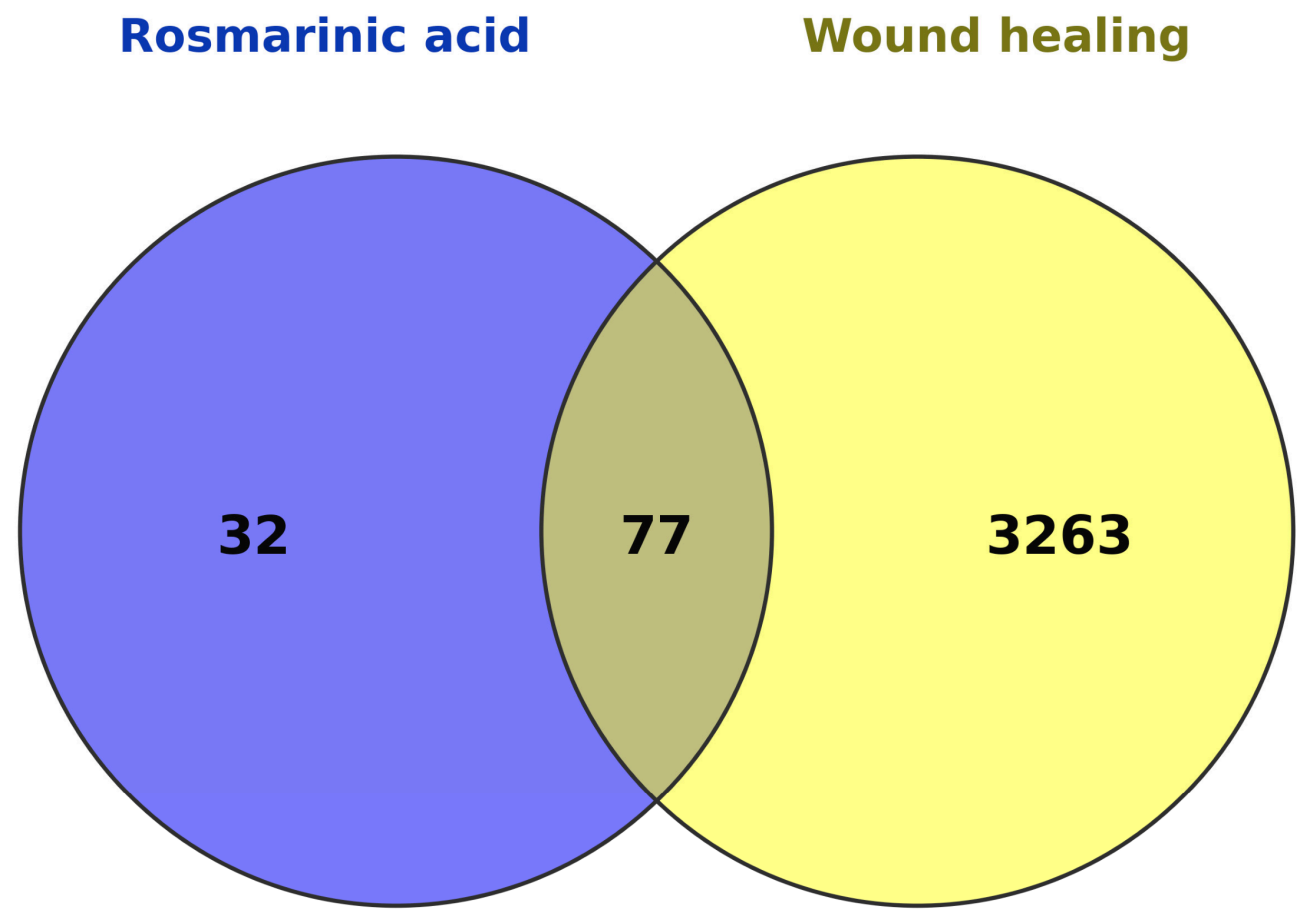
A Venn diagram illustrating the overlapping target genes between RA-related targets and wound healing-associated genes.

Potential targets of RA were retrieved from the TCMSP, STITCH, CTD, and SwissTargetPrediction databases. After data integration and removal of duplicate entries, 109 unique targets were retained. All targets were standardized to official gene symbols using the UniProt database to ensure nomenclature consistency (Supplementary Material S3, Table S1). Genes associated with wound healing were collected from the GeneCards, MalaCards, and OMIM databases. After integration and removal of duplicate entries, a total of 3.352 wound healing-related genes were obtained (Supplementary File S3). The intersection between RA-related targets and wound healing-associated genes was determined using Venn diagram analysis, resulting in 77 overlapping targets (Figure 8 and Supplementary File S3).

#### 2.5.3. PPI Network and Hub Gene Analysis

The 77 intersected target genes were imported into the STRING database (version 12.0; https://string-db.org/) to construct a protein–protein interaction (PPI) network. The organism was restricted to *Homo sapiens*, and the minimum required interaction score was set to (0.7).

**Figure 9:**
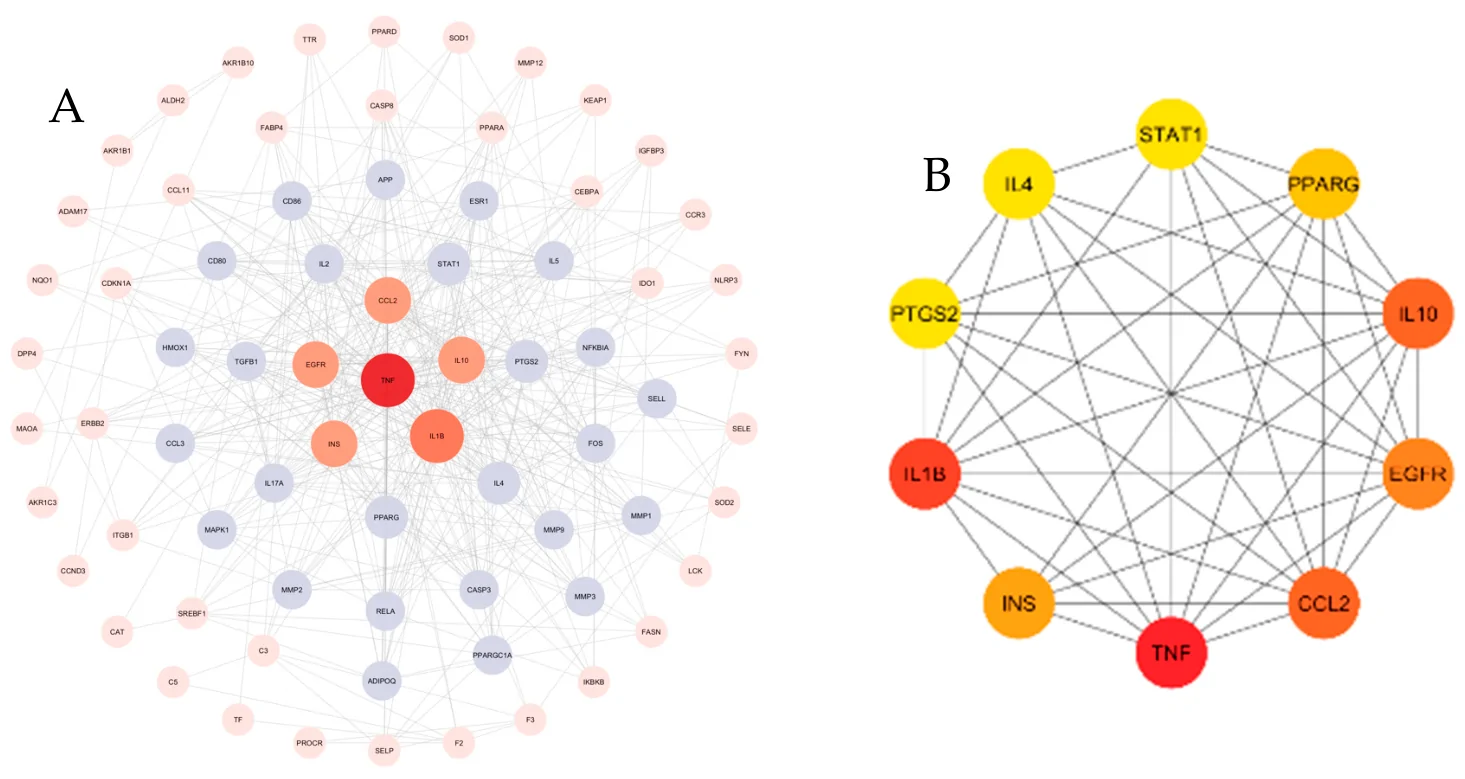
Interaction network of shared targets for RA and wound healing (**A**); interaction network of key targets for RA against wound healing (**B**).

After removing disconnected nodes, the resulting PPI network consisted of 76 nodes and 415 edges, with an average node degree of 10.9. These parameters indicate a densely interconnected network among the candidate targets. The PPI network generated from STRING was subsequently imported into Cytoscape (version 3.10.4) for visualization and topological analysis. NetworkAnalyzer was employed to calculate key network parameters, particularly degree centrality, in order to identify hub genes within the network. The complete PPI network is presented in Figure 9, where node size and color intensity were mapped according to degree values to reflect the relative importance of each target. Nodes with higher connectivity were represented by larger sizes and warmer colors.

Based on the ranking of degree values, the top 10 hub genes were identified as tumor necrosis factor (TNF), interleukin-1 beta (IL-1β), interleukin-10 (IL-10), C-C motif chemokine ligand 2 (CCL2), epidermal growth factor receptor (EGFR), insulin (INS), peroxisome proliferator-activated receptor gamma (PPAR-γ), interleukin-4 (IL-4), prostaglandin-endoperoxide synthase 2 (PTGS2), and signal transducer and activator of transcription 1 (STAT1).

A subnetwork comprising these 10 hub genes is shown in Figure 9. The detailed ranking of the hub genes, including their protein names and degree scores, is summarized in Table 6. These highly connected nodes may represent central regulatory components involved in the wound healing process.

#### 2.5.4. Functional Enrichment Analysis

**Figure 10:**
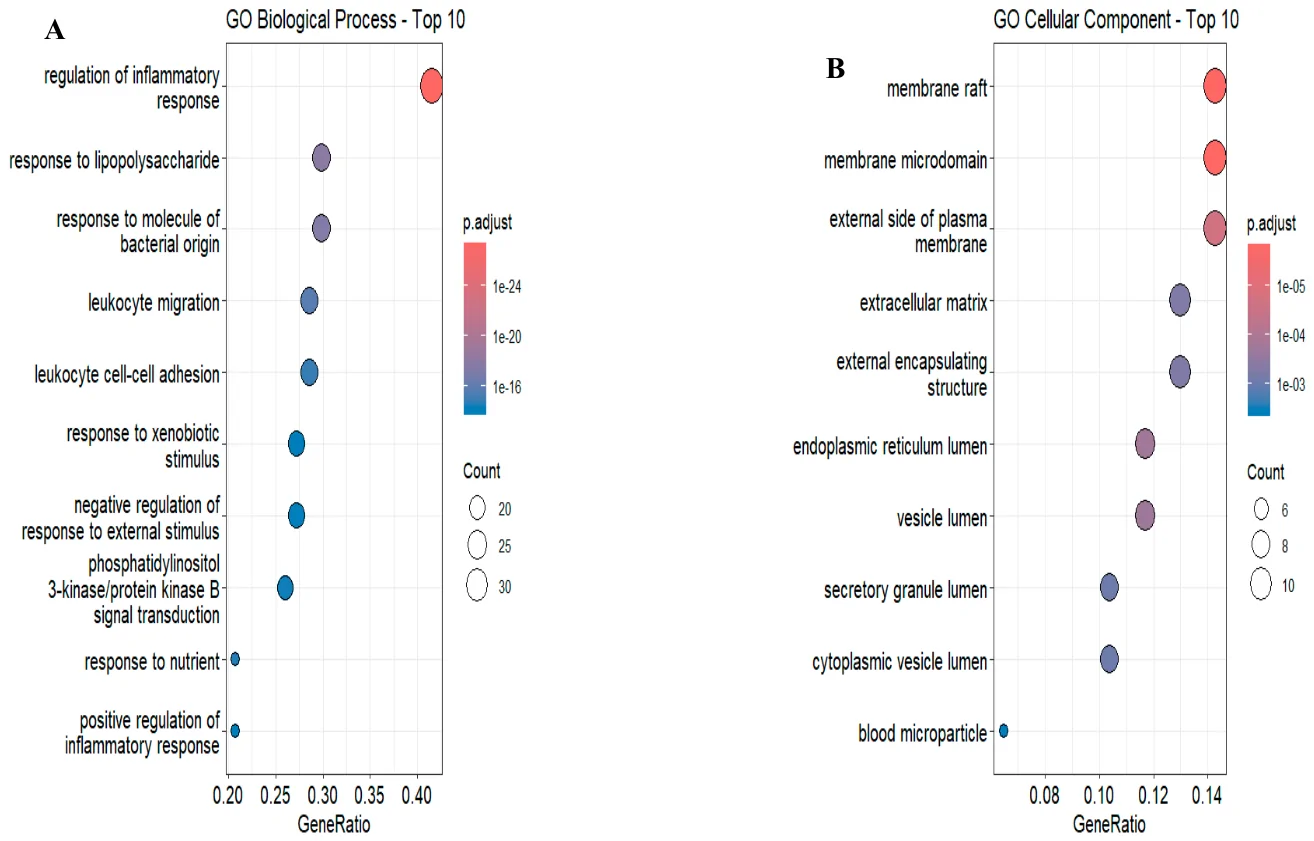

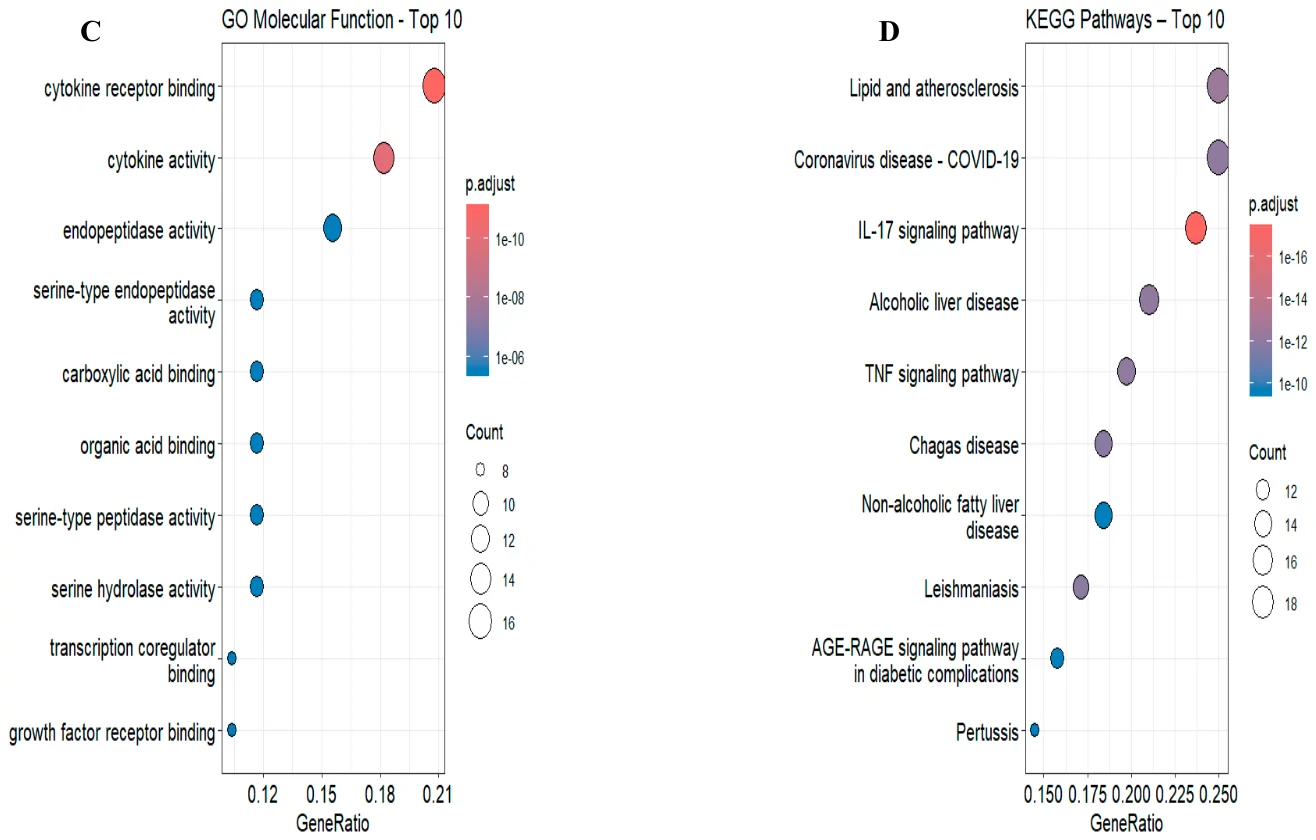
GO and KEGG enrichment analysis of candidate targets. (**A**) GO biological process enrichment analysis. (**B**) GO cellular component enrichment analysis. (**C**) GO molecular function enrichment analysis. (**D**) KEGG pathway enrichment analysis. Bubble size represents gene count, and color intensity corresponds to adjusted *p*-value.

To further elucidate the biological characteristics of the candidate targets, Gene Ontology (GO) and Kyoto Encyclopedia of Genes and Genomes (KEGG) pathway enrichment analyses were performed. The top 10 enriched terms were visualized according to gene ratio, gene count, and adjusted *p*-value (*p*.adjust) (Figure 10).

***GO Biological Process (BP) Enrichment Analysis*** 

As shown in Figure 10, GO-BP enrichment analysis demonstrated that the candidate targets were predominantly involved in inflammation- and immune-related processes. The most significantly enriched term was regulation of inflammatory response, followed by: response to lipopolysaccharide, response to molecule of bacterial origin, leukocyte migration, leukocyte cell–cell adhesion, response to xenobiotic stimulus, negative regulation of response to external stimulus, and phosphatidylinositol 3-kinase/protein kinase B signal transduction (Supplementary File S4). These results indicate that the predicted targets are mainly associated with inflammatory regulation, immune activation, and PI3K/AKT-related signaling pathways, suggesting a central role in immune-mediated biological responses.

***GO Cellular Component (CC) Enrichment Analysis*** 

The GO-CC analysis revealed that the targets were primarily localized to membrane-associated and extracellular regions. Significantly enriched cellular components included: membrane raft, membrane microdomain, external side of plasma membrane, extracellular matrix, external encapsulating structure, endoplasmic reticulum lumen, vesicle lumen, and secretory granule lumen (Supplementary File S5). These findings suggest that the targets are mainly distributed in membrane microdomains and extracellular compartments, indicating their involvement in signal transduction, secretion, and ECM-related biological functions.

***GO Molecular Function (MF) Enrichment Analysis*** 

GO-MF enrichment analysis indicated that the targets were mainly enriched in cytokine- and protease-related molecular functions. The most enriched terms included: cytokine receptor binding, cytokine activity, endopeptidase activity, serine-type endopeptidase activity, serine-type peptidase activity, serine hydrolase activity, and growth factor receptor binding (Supplementary File S6). These results suggest that the targets may regulate inflammatory signaling and immune responses through cytokine interactions and proteolytic activities.

***KEGG Pathway Enrichment Analysis*** 

KEGG pathway enrichment analysis demonstrated that the candidate targets were significantly enriched in inflammation- and infection-related pathways. The top enriched pathways included: the IL-17 signaling pathway, the TNF signaling pathway, the AGE-RAGE signaling pathway in diabetic complications, lipids and atherosclerosis, Coronavirus disease (COVID-19), alcoholic liver disease, and non-alcoholic fatty liver disease (Supplementary File S7). Notably, the IL-17 and TNF signaling pathways showed high gene ratios and strong statistical significance, highlighting their potential central role in the underlying mechanism. These findings suggest that the predicted targets of RA may influence inflammatory, immune-related, and metabolic pathways potentially relevant to wound healing.

#### 2.5.5. The “Drug–Target–Pathway–Disease” Network

**Figure 11:**
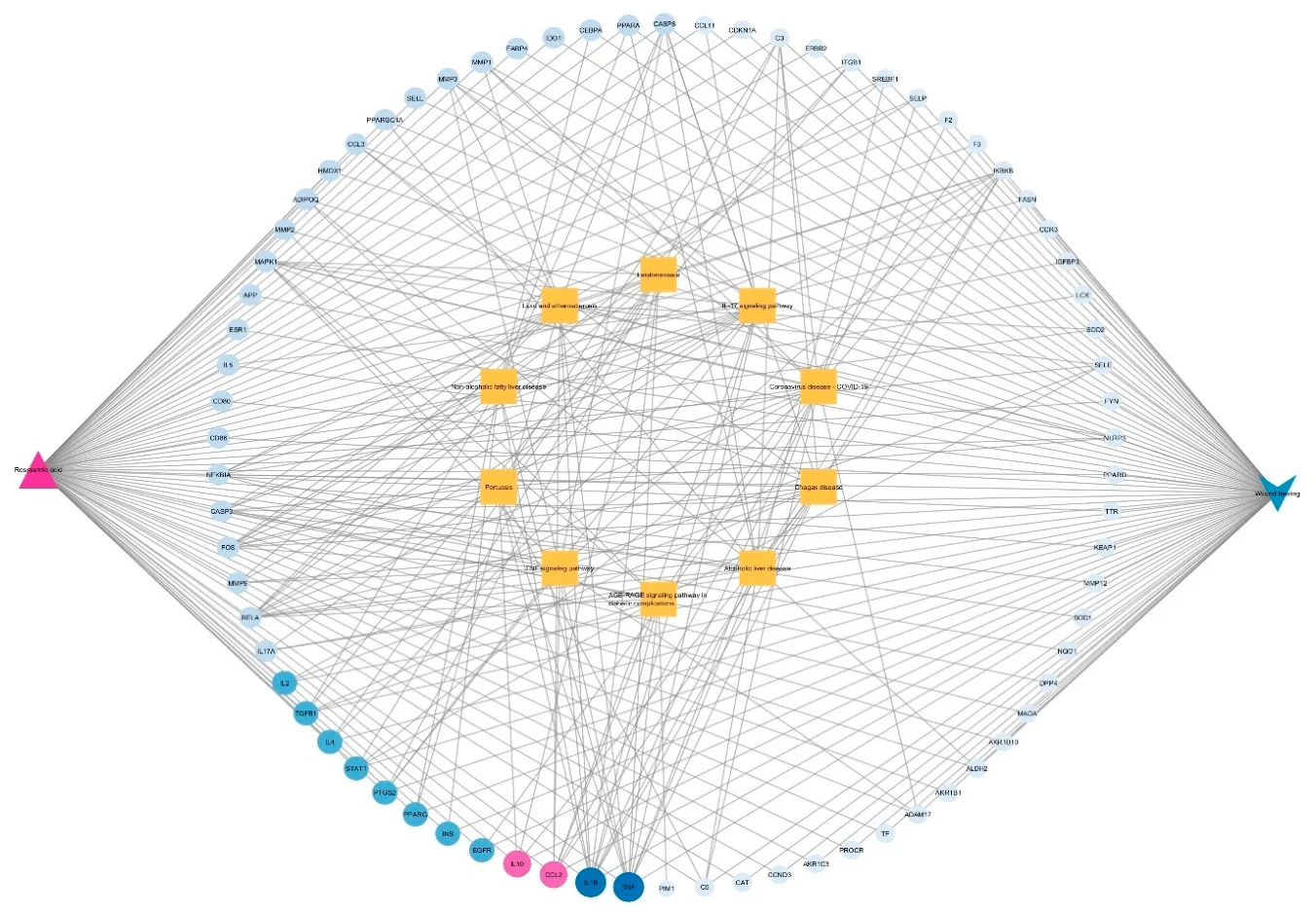
The drug–target–pathway–disease interaction network. The triangular node represents RA, circular nodes represent key targets, square nodes represent the top 10 enriched KEGG pathways, and the diamond node represents wound healing. The edges indicate the interactions among the compound, targets, pathways, and disease.

A network diagram was constructed using Cytoscape 3.10.4 software to illustrate the relationships among RA, the 73 key targets (including genes with degree ≥ 2), the top 10 enriched signaling pathways, and wound healing (Figure 11).

In the present study, a network pharmacology approach was employed to explore the potential mechanisms by which RA may promote wound healing. Through PPI network construction, hub gene identification, functional enrichment analysis, and drug–target–pathway–disease network modeling, these findings provide a systems-level estimation of the potential molecular interactions of RA in wound healing. The PPI network demonstrated a high level of connectivity among the intersected targets, indicating strong functional associations between these proteins. The identification of hub genes based on degree centrality highlighted several key regulators, including TNF, IL1*β*, IL10, CCL2, EGFR, INS, PPARG, IL4, PTGS2, and STAT1. These genes are widely recognized as critical mediators of inflammatory signaling, immune modulation, and tissue repair. TNF and IL1*β* are major pro-inflammatory cytokines involved in the initiation and amplification of inflammatory responses [42,43], while IL10 and IL4 function as anti-inflammatory cytokines that contribute to inflammation resolution and tissue regeneration [44]. PTGS2 is associated with prostaglandin synthesis and inflammatory mediation [45], whereas STAT1 plays an essential role in cytokine-driven signal transduction [46]. EGFR and INS are closely related to cell proliferation, migration, and metabolic regulation, processes essential for the proliferative phase of wound healing [47,48]. The central positions of these genes within the predicted network suggest that they may represent potential regulatory nodes relevant to its biological effects. However, these interactions obtained via computational predictions require experimental validation. The GO enrichment analysis suggested that the candidate targets were predominantly involved in biological processes related to inflammatory response, leukocyte migration, response to bacterial stimuli, and PI3K/AKT signal transduction. These processes are fundamental to the early inflammatory phase of wound repair and subsequent transition to tissue regeneration. The enrichment of cytokine receptor binding and cytokine activity in the molecular function category supports the notion that RA may modulate cytokine-mediated communication between immune and resident cells. In addition, the cellular component analysis indicated that many targets are localized in membrane rafts, extracellular regions, and secretory structures, which are key sites for receptor signaling and intercellular communication during tissue repair. KEGG pathway analysis showed significant enrichment in the IL-17 and TNF signaling pathways, both of which are associated with inflammatory signaling activation. Overactivation of these pathways is associated with excessive inflammation and impaired wound healing. Therefore, modulation of IL-17 and TNF signaling may represent a plausible hypothesis obtained from network pharmacology analysis, but this hypothesis requires experimental confirmation. The IL-17 signaling pathway is known as an important regulator of wound healing as it coordinates inflammation and tissue repair. Physiological IL-17 activity contributes to host defense, angiogenesis, fibroblast activation, and epithelial repair, while sustained activation leads to excessive inflammation, neutrophil recruitment, extracellular matrix disruption via increased matrix metalloproteinase expression, and delayed wound healing. Therefore, maintaining a balanced IL-17 response is essential for successful tissue repair. In this study, the identification of the IL-17 signaling pathway via network pharmacology suggests that RA may contribute to wound healing, at least in part, through the modulation of inflammatory responses associated with this pathway [49]. The enrichment of the AGE–RAGE signaling pathway in diabetic complications further suggests potential relevance in chronic or diabetic wounds, where oxidative stress and persistent inflammation are prominent pathological features. The AGE-RAGE signaling pathway has been reported to contribute to impaired wound healing, especially under diabetic conditions. Excessive accumulation of advanced glycation end products activates RAGE in macrophages, leading to persistent inflammation, defective phagocytosis, impaired M1-M2 macrophage transition, and decreased secretion of pro-repair growth factors such as VEGF and PDGF. Consequently, the inflammatory phase is prolonged, and tissue regeneration may be delayed. In this study, identification of the AGE-RAGE signaling pathway in RAGE pharmacology analysis suggests that RA may contribute to wound healing through the modulation of macrophage-mediated inflammatory responses associated with this pathway [50]. Additional enrichment in pathways related to lipid metabolism and inflammatory diseases indicates that RA may also influence metabolic–inflammatory interactions that impact tissue repair. The constructed drug–target–pathway–disease network visually demonstrates the multi-target and multi-pathway characteristics of RA. By focusing on genes with a degree ≥ 2, the analysis emphasized targets with stronger network connectivity and potential biological significance. Overall, the network pharmacology analysis suggests that RA may be relevant to multiple interconnected inflammatory and immune signaling pathways in wound healing. Nevertheless, these findings are based exclusively on in silico analyses and further molecular, cellular, and in vivo studies are required to verify the predicted targets and signaling pathways.

## 3. Materials and Methods

### 3.1. Chemicals

The chemical materials used in the biological activity studies, ethanol, hydrochloric acid (HCl), HPLC-grade methanol, dimethyl sulfoxide (DMSO) tris, sodium chloride (NaCl), calcium chloride (CaCl_2_), potassium chloride (KCl), sodium hydroxide (NaOH), sodium borate, dimethylaminobenzaldehyde, N-[3-(2-Furyl)acryloyl]-Leu-Gly-Pro-Ala, sodium lauryl sulfate, 3-(4,5-dimethylthiazol-2-yl)-2,5-diphenyltetrazolium bromide (MTT), N-succinyl-ala-ala-ala-p-nitroanilide, *Clostridium histolyticum* collagenase, hyaluronidase from bovine testes, elastase from human neutrophil, catechin, epigallocatechin gallat, and hyaluronic acid sodium salt, were purchased from Sigma-Aldrich (St. Louis, MO, USA). Dulbecco’s Modified Eagle Medium (DMEM), phosphate-buffered saline (PBS), fetal bovine serum (FBS), penicillin-streptomycin solution, and trypsin-EDTA (TRY-3B) were obtained from Capricorn (Ebsdorfergrund, Germany). NCTC clone 929 (L929 mouse fibroblast cells, connective mouse tissue) were obtained from ATCC (CCL-1) (Manassas, VA, USA). RA was previously isolated from the plant *T. orientalis* in our studies [16,17].

In this study, a magnetic stirrer (MR, Heidolph Instruments, Schwabach, Germany), refrigerator (Arçelik, Istanbul, Türkiye), evaporator (Heidolph), precision balance (Ohaus Pioneer, Parsippany, NJ, USA), incubator (Memmert, Schwabach, Germany), pH meter (Hanna Instruments, Woonsocket, RI, USA), centrifuge (Elektromag, Istanbul, Türkiye), spectrophotometer (SPECTROstar Nano, BMG LABTECH, Ortenberg, Germany), vortex (Reax Top, Heidolph), freeze dryer (Martin Christ, Osterode am Harz, Germany), microwave oven (START SYNTH, Milestone, Sorisole, Italy), ultrasonic water bath (İsolab, Eschau, Germany), HPLC (Shimadzu, Kyoto, Japan), CO_2_ incubator (HF90/HF240, Heal Force, Shanghai, China), inverted microscope (DMi1, Leica Microsystems, Wetzlar, Germany), centrifuge (NF400 Classic 212, NUVE, Ankara, Türkiye), biological safety cabinet (Safefast, Faster, Cornaredo, Italy), and automated cell counter (Thermo Fisher Scientific, Waltham, MA, USA) were used.

### 3.2. Plant Material

The flowering aerial parts of *T. orientalis* were collected and identified in Rize in Türkiye by Prof. Ufuk ÖZGEN using Flora of Turkey and the East Aegean Islands (Vol. 6) [51]. The voucher specimen (No. KATO 15486) was stored at the KATO Herbarium (Karadeniz Technical University, Faculty of Forestry), Trabzon, Türkiye. The flowering aerial parts were selected based on our previous phytochemical investigations, which demonstrated that these plant parts are particularly rich in RA and that RA was successfully isolated [16,51]. The species was shade-dried for approximately one week at room temperature (25 ± 2 °C) and ground using a laboratory mill to obtain a homogeneous powder prior to extraction. The dried and powdered aerial parts (leaves, stem and flowers) of the species were used in 20 g portions during the extraction process.

### 3.3. Extraction Procedures and Experimental Design

#### 3.3.1. Preparation of the Conventional Maceration Extract

The dried and powdered flowering aerial parts of *T. orientalis* (50 g) were macerated with 200 mL of ethanol at 25 °C. The extraction was performed in triplicate. The extracts were filtered, and the solvent was removed to dryness using an evaporator.

#### 3.3.2. Fractional Factorial Screening Design and Box–Behnken Optimization

The experimental design was created using the fractional factorial design approach with the Design Expert 11.1.2 (Stat-Ease, Minneapolis, MN, USA) software, including six parameters. The extraction variables were selected based on preliminary experiments and their reported influence on microwave-assisted extraction efficiency by controlling energy input, target temperature, and mass [52]. The effects of the factors on the system were statistically evaluated using the same software. During the design phase, two levels (minimum and maximum) were determined for each factor. The number of experiments was planned to be n parameters, and 2^n−1^ experiments were performed in accordance with the fractional factorial approach. In this study, extraction time, extraction temperature, solvent volume, microwave power, ultrasonic duration, and vortex duration were selected as independent variables. The minimum and maximum levels of these variables are presented in Table 7.

The experimental design created to determine the parameters that have the greatest impact on the extraction process is presented in Table 8. For each experimental run, 20 g of the dried and powdered plant material was extracted with ethanol under the conditions specified in Table 8. The resulting extracts were filtered, and the solvent was removed to dryness using a rotary evaporator.

The RA content of the extracts obtained according to Table 8 was analyzed using reverse-phase high-performance liquid chromatography (RP-HPLC). RSM was applied to optimize the extraction conditions. In the optimization phase, a Box–Behnken experimental design with three independent variables and five replicates at the central point was used. Experiments were conducted in a random order, with a total of 17 experiments. Multiple regression analysis was applied to the obtained data, and the statistical fit of the model was evaluated using ANOVA. The resulting experimental design matrix is presented in Table 9.

The extraction process was optimized using a Box–Behnken design with RA as the response variable. Based on the response surface analysis, the optimal conditions selected for maximizing RA yield were a microwave extraction time of 60 s, an extraction temperature of 50 °C, and a solvent volume of 10 mL. TO-O used in the subsequent chemical and biological experiments was prepared under these selected conditions.

### 3.4. RP-HPLC Analysis

#### 3.4.1. Determination of Rosmarinic Acid Quantity

The RA content of the extracts was analyzed using a modified method based on the study by Ergin et al. [53]. Dry extracts (10 mg/mL) and RA standard solutions (5–500 µg/mL) were prepared with HPLC-grade methanol and filtered through 0.45 µm membrane filters. Chromatographic analyses were performed using the gradient elution method, with mobile phase A consisting of 100% methanol and mobile phase B consisting of a 2% aqueous acetic acid solution at pH 2.7. Analyses were carried out using an SPD-M20A Diode Array Detector (DAD) with a C18 column (RP-C18, 150 mm × 4.6 mm, 5 µm). The column temperature was kept constant at 25 °C, and the flow rate was set at 1.5 mL/min. The injection volume was 10 µL, and the detection wavelength was set at 280 nm. The analytical method was validated according to the ICH Q2 (R2) guideline. The validation included the assessment of linearity, accuracy, precision, specificity, LOD, and LOQ. A summary of the validation parameters together with representative chromatograms and detailed validation results is provided in Supplementary File S1 (Table S1).

#### 3.4.2. Targeted RP-HPLC Analysis of the Rosmarinic Acid-Enriched Extract

Targeted RP-HPLC analysis was performed to determine RA together with selected compounds including gallic acid, catechin, *p*-hydroxybenzoic acid, vanillic acid, ferulic acid, *p*-coumaric acid, sinapic acid, and quercetin in TO-O using the chromatographic conditions described in Section 3.4.1. For this analysis, 4.4 mg of the dry extract was dissolved in 10 mL of HPLC-grade methanol and filtered through a 0.45 µm membrane filter. The quantified concentrations were normalized based on the amount of dry extract used, and expressed as mg/g dry extract.

### 3.5. Enzyme İnhibition Studies

The collagenase inhibitory effect was determined using a spectrophotometric method [2]. A 96-well plate was used in the experiment. Test solutions (25–200 µg/mL), enzyme (*Clostridium histolyticum* collagenase (0.8 U/mL)) and substrate (N-[3-(2-Furyl)acryloxyl]-Leu-Gly-Pro-Ala (2 mM)) were prepared in 50 mM Tris buffer (0.4 M NaCl and 0.01 M CaCl_2_, pH 7.5). Next, 25 µL of buffer solution, 25 µL of test solution, and 25 µL of enzyme solution were added and incubated at 37 °C for 15 min. Then, 50 µL of substrate was added and incubated for 15 min at 37 °C. Absorbance values were read at 340 nm using a spectrophotometer. Catechin was used as a positive control. The experiments were performed in triplicate, and the necessary calculations were carried out based on the average absorbance value.

Elastase inhibition was investigated based on the method of Moon et al. [54]. A 96-well plate was used in the experiment. Test solutions (25–200 µg/mL), enzyme (human neutrophil elastase enzyme (17 mU/mL)) and substrate (N-succinyl-ala-ala-ala-p-nitroanilite (5 mM)) were prepared in 0.2 M Tris-HCl buffer (pH 7.8). Next, 50 µL of buffer solution, 50 µL of test solution, and 100 µL of enzyme solution were added and incubated at 37 °C for 15 min. Then, 50 µL of substrate was added and incubated for 30 min at 37 °C. Absorbance values were read at 410 nm using a spectrophotometer. Epigallocatechin gallate (EGCG) was used as a positive control. The experiments were performed in triplicate, and the necessary calculations were carried out based on the average absorbance value.

The inhibition of the hyaluronidase enzyme was evaluated by measuring the amount of N-acetylglucosamine released from sodium hyaluronate. A 96-well plate was used in the experiment. Test solutions (25–200 µg/mL), enzyme (bovine hyaluronidase, 4200 U/mL) and substrate (sodium hyaluronate, 1.2 mg/mL) were prepared in 0.1 M acetate buffer (pH 3.6). Next, 50 µL of test solution and 50 µL of enzyme solution were added and incubated at 37 °C for 20 min. Then, 20 µL of CaCl_2_ (12.5 mM) was added to the mixture and incubated for 10 min at 37 °C. Next, 50 µL of sodium hyaluronate (1.2 mg/mL) was added and incubated at 37 °C for 40 min. To the resulting mixture, 10 µL of NaOH (0.9 M) and 10 µL of sodium borate (0.2 M) were added and incubated in a boiling water bath for 3 min. After cooling the mixture to room temperature, 50 µL of *p*-dimethylaminobenzaldehyde was added and incubated at 37 °C for 10 min until a color developed. Finally, the absorbance of the mixture was measured at 585 nm. Tannic acid was used as a positive control. The experiments were performed in triplicate and the necessary calculations were carried out based on the average absorbance value [55,56].

The inhibitory effects of test solutions and standards on collagenase, elastase, and hyaluronidase enzymes were calculated according to the following equation:

$$\%\;\mathrm{Inhibition}=[\frac{\mathrm{Acontrol}-\mathrm{Asample}}{\mathrm{Acontrol}}]\times 100$$

*A_control_* is the absorbance of the uninhibited control reaction containing the enzyme and substrate but no active test sample.

*A_sample_* is calculated by subtracting the absorbance of the sample blank containing the test sample, enzyme, and buffer from the mean absorbance of the replicate reaction wells containing the test sample, enzyme, and substrate.

### 3.6. Cell Culture Studies

Mouse fibroblast (L929) cells (passages 6–10) were cultured in DMEM supplemented with 10% fetal bovine serum and 1% penicillin/streptomycin at 37 °C in a humidified atmosphere containing 5% CO_2_. The cells were passaged at approximately 90% confluence using trypsin-EDTA as previously described [57]. The cytotoxicity of the extracts was evaluated using the MTT assay, which is based on the reduction of tetrazolium salt to insoluble purple formazan crystals by mitochondrial enzymes, NADH-dependent succinate dehydrogenase, in viable cells [58,59]. The extracts were prepared as stock solutions in methanol (100 mg/mL) and further diluted with culture medium to obtain final concentrations of 0.25, 2.5, 25, and 250 µg/mL. L929 cells were seeded into 96-well plates (1 × 10^4^ cells/well) and incubated for 24 h. The cells were then treated with the extracts at the indicated concentrations for 24 h. Cells treated with 0.5% (*v*/*v*) methanol and sodium lauryl sulfate (0.3 mM) served as negative and positive controls, respectively. After treatment, MTT solution (0.5 mg/mL) was added and incubated for 3 h. The resulting formazan crystals were dissolved in DMSO, and absorbance was measured at 570 nm with a reference wavelength of 690 nm [59]. Cell viability was expressed as a percentage relative to the solvent control, and the results were presented as mean ± standard deviation.

The scratch assay was performed using 12-well culture plates. L929 cells were seeded at a density of 1.2 × 10^5^ cells per well and incubated for 24 h until 90–100% confluence. Linear scratch wounds were created using a sterile 200 µL pipette tip. Detached cells were removed, and fresh medium containing the non-cytotoxic concentration of the samples was added. Wells containing untreated cells maintained in culture medium without any test sample served as the control. Cells were incubated at 37 °C in a humidified atmosphere with 5% CO_2_, and images of the wound area were captured at 0, 6, 12, and 18 h. Wound closure was quantified by calculating the percentage of the closed area using Image J software, based on cell migration into the scratched region [57].

### 3.7. Statistical Analysis

All samples were analyzed in triplicate, and the experimental data are reported as mean ± standard deviation (SD). IC_50_ values, % viability and % wound closure were determined with Microsoft Excel. Statistical differences were assessed using one-way ANOVA and two-way ANOVA, followed by Tukey’s multiple comparisons test, utilizing GraphPad Prism 8.0.2 (GraphPad Prism Software, San Diego, CA, USA).

### 3.8. In Silico Target Prediction and Network Pharmacology Analysis of Rosmarinic Acid

**Figure 12:**
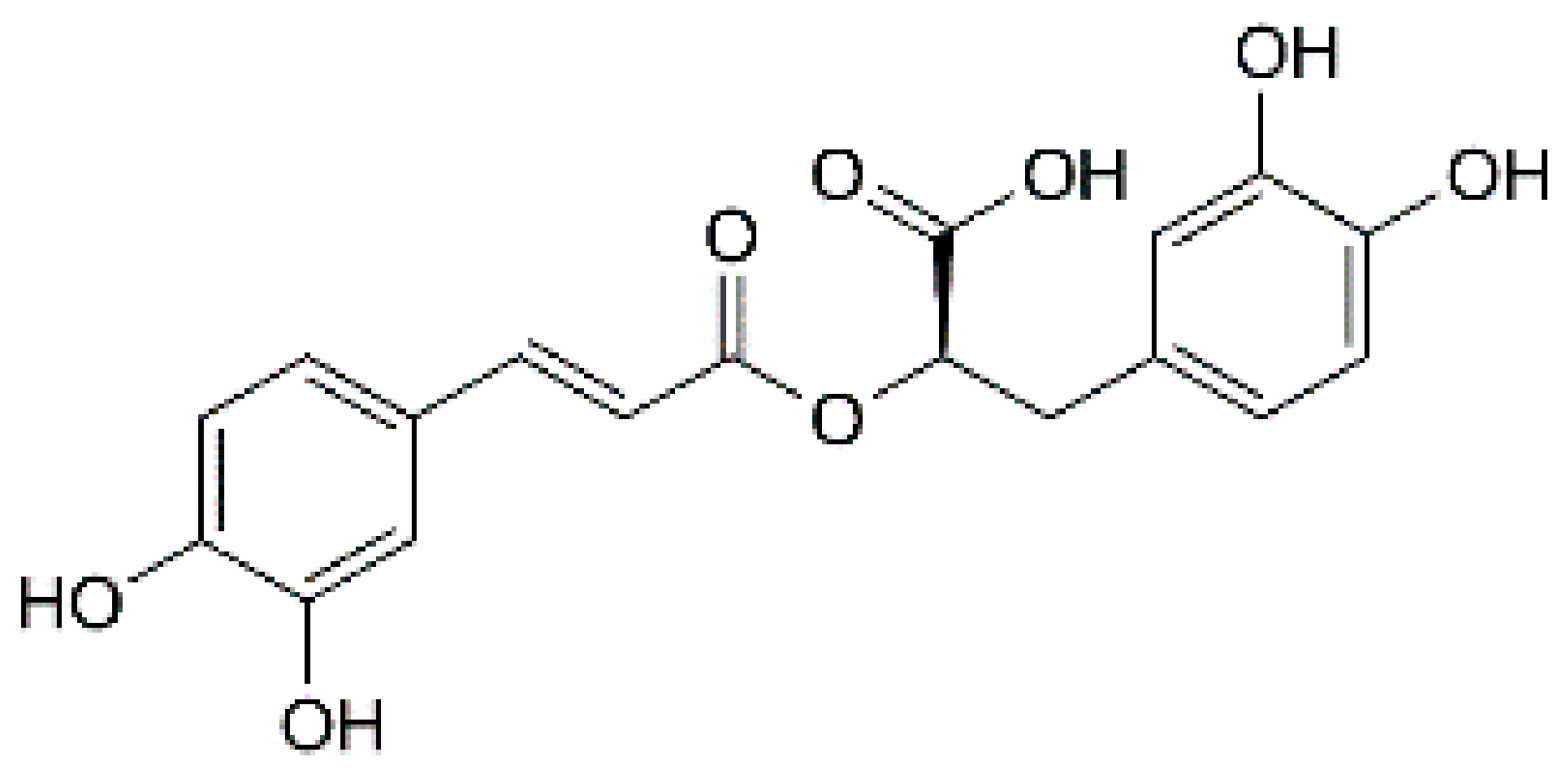
The structural formula of RA.

The network pharmacology analysis was performed using RA (Figure 12) because the extraction process was optimized based on its content; previous phytochemical studies have consistently identified it as one of the predominant phenolic constituents of the species, and its wound healing properties have been well documented in the literature. Therefore, the analysis was planned to explore the potential molecular mechanisms associated with RA rather than to represent the pharmacological profile of the extract.

#### 3.8.1. Common Targets Prediction of Rosmarinic Acid

Potential targets of the selected compound were retrieved from multiple public databases, including the Traditional Chinese Medicine Systems Pharmacology Database and Analysis Platform (TCMSP, https://tcmsp-e.com/tcmsp.php, accessed on 6 April 2026) [60], Search Tool for Interactions of Chemicals STITCH (http://stitch.embl.de/cgi/, accessed on 6 April 2026) [61], the Comparative Toxicogenomics Database (CTD, http://ctdbase.org/, accessed on 6 April 2026) [62] and SwissTargetPrediction (http://www.swisstargetprediction.ch/, accessed on 6 April 2026) [63]. All collected targets were imported into the UniProt database (https://www.uniprot.org/, accessed on 6 April 2026) [64] to standardize protein names and convert them into official gene symbols. Duplicate entries were removed to generate a non-redundant list of potential targets for subsequent network analysis.

#### 3.8.2. Identification of Wound Healing-Related Target Genes

Genes associated with wound healing were retrieved from the GeneCards (https://www.genecards.org/, accessed on 6 April 2026) [65], MalaCards (https://www.malacards.org/, accessed on 6 April 2026) [66] and OMIM (https://www.ncbi.nlm.nih.gov/omim, accessed on 6 April 2026) [67] databases using the keywords “wound healing” and “cutaneous wound.” In GeneCards, only genes with a relevance score above the median were retained as the inclusion criterion. All genes obtained from the three databases were integrated, and duplicate entries were removed to generate a non-redundant set of wound-related targets. Subsequently, the predicted targets of RA compound were intersected with the wound-related gene set using the Venny 2.1.0 online tool (https://bioinfogp.cnb.csic.es/tools/venny/, accessed on 6 April 2026). The overlapping genes were considered potential therapeutic targets involved in the wound healing process.

#### 3.8.3. Protein–Protein Interaction (PPI) Network Construction and Core Target Screening

The intersected target genes were imported into the STRING database (version 12.0) (https://string-db.org/, accessed on 6 April 2026) for protein–protein interaction (PPI) network construction. The organism parameter was limited to *Homo sapiens*, and the minimum required interaction score was set to a high-confidence threshold (0.7). Under these conditions, a protein–protein interaction (PPI) network was constructed to assess the functional associations among the candidate targets. The fundamental topological properties of the network, including the total number of nodes and edges, were subsequently extracted to characterize its structural features and interaction density.

The interaction data exported from STRING were then imported into Cytoscape software (version 3.10.4) [68] for network visualization and topological analysis. Network topology parameters were calculated, and core targets were identified by ranking nodes according to degree value. The top 10 nodes with the highest degree values were defined as hub genes and used for subsequent analyses.

#### 3.8.4. ADME and Toxicity Prediction

The absorption, distribution, metabolism, and excretion profile of RA was predicted using SwissADME (http://www.swissadme.ch, accessed on 6 April 2026), an online platform for evaluating pharmacokinetic behavior and drug-likeness [69]. The canonical SMILES of the stereochemically defined natural form of RA was used as input, and physicochemical descriptors including molecular weight, lipophilicity (LogP), topological polar surface area (TPSA), and Lipinski compliance were calculated.

Toxicological properties were estimated using ProTox-3.0 (https://tox.charite.de/protox3, accessed on 6 April 2026) [70]. The model predicted acute oral toxicity (LD50 and toxicity class) and organ-specific endpoints including hepatotoxicity, neurotoxicity, nephrotoxicity, respiratory toxicity, and cardiotoxicity. Additional endpoints—mutagenicity, carcinogenicity, cytotoxicity, immunotoxicity, BBB permeability, ecotoxicity, and clinical toxicity—were evaluated using machine-learning-based structural similarity algorithms.

#### 3.8.5. Functional Enrichment Analysis

The intersected target genes were analyzed using RStudio (version 2026.1.0.392). Gene symbols were initially converted to Entrez Gene IDs using the org.Hs.eg.db. Subsequently, functional enrichment was conducted with the clusterProfiler package [62,71] including Gene Ontology (GO) analysis for biological process (BP), molecular function (MF), and cellular component (CC), along with Kyoto Encyclopedia of Genes and Genomes (KEGG) pathway enrichment. Statistical significance was defined as *p* < 0.05 and false discovery rate (FDR, q-value) < 0.05 based on Benjamini–Hochberg correction. Finally, the top ten GO terms from each category and the top ten KEGG pathways were visualized using dot plots generated with the enrichplot and ggplot2 packages [72].

#### 3.8.6. Establishing a “Drug–Target–Pathway–Disease” Network

A “drug–target–pathway–disease” network was constructed using Cytoscape (version 3.10.4) to visualize the inter-relationships among these entities. This network highlights connections between key targets and their associated pathways. Only the top 10 KEGG pathways, ranked by the lowest adjusted *p*-values, were incorporated; targets lacking associations with these pathways were excluded from the analysis.

## 4. Conclusions

The present study demonstrated the in vitro potential wound healing properties of TO-O obtained from *T. orientalis* growing in Türkiye. Optimization of the extraction process using RSM identified solvent volume, temperature, and extraction time as key factors influencing RA yield. The optimized extract exhibited inhibitory activity against collagenase, elastase, and hyaluronidase, enzymes involved in ECM degradation. In addition, it showed no cytotoxicity on L929 fibroblasts within the tested concentration range, and promoted wound closure in the scratch assay. Collectively, these findings indicate that *T. orientalis* represents a promising natural source for wound healing applications.

Network pharmacology analysis provided preliminary mechanistic insights through a computational prediction approach, identifying putative molecular targets and signaling pathways potentially associated with the wound healing activity of RA. However, these findings are based solely on computational predictions and should not be interpreted as experimentally validated molecular mechanisms. Furthermore, although RA was selected as the marker compound for extraction optimization, the observed biological activity cannot be attributed exclusively to this compound. Other identified and chemically uncharacterized compounds in the extract may also contribute to the overall biological activity.

The present study has several limitations. Since no proliferation inhibitor was included in the scratch assay, the observed wound closure may reflect the combined effects of fibroblast migration and proliferation. In addition, the pharmacokinetic profile, metabolism, oral bioavailability, and in vivo wound healing effect of the samples were not investigated. Future studies should therefore focus on comprehensive phytochemical characterization of the extract, molecular validation of the predicted targets and signaling pathways, pharmacokinetic characterization, formulation development, and evaluation in appropriate in vivo wound healing models.

## Figures and Tables

**Table 1:** The ANOVA results of the reduced fractional factorial mode.

Source	Sum of Squares	Mean Square	F-Value	*p*-Value	
Model	1.038 × 10^6^	1.483 × 10^5^	22.84	<0.0001	Significant
A-MW Extraction Time	461.71	461.71	0.0711	0.7920	
B-Extraction Temperature	13,790.02	13,790.02	2.12	0.1579	
C-Solvent Volume	9.245 × 10^5^	9.245 × 10^5^	142.43	<0.0001	
Residual	1.558 × 10^5^	6491.12			
Cor Total	1.194 × 10^6^				

**Table 2:** The ANOVA results of the quadratic model in the Box–Behnken design.

Source	Sum of Squares	Mean Square	F-Value	*p*-Value	
Model	788.76	87.64	37.82	<0.0001	significant
A-MW Extraction Time	0.0147	0.0147	0.0063	0.9388	
B-Extraction Temperature	62.71	62.71	27.07	0.0012	
C-Solvent Volume	636.43	636.43	274.68	<0.0001	
C^2^	61.13	61.13	26.38	0.0013	
Residual	16.22	2.32			
Lack of Fit	7.60	2.53	1.18	0.4234	not significant

**Table 3:** Box–Behnken suitability data.

Source	Sequential *p*-Value	Lack-of-Fit *p*-Value	Adjusted R^2^	Predicted R^2^	
Linear	<0.0001	0.0676	0.8382	0.7397	
2FI	0.5160	0.0535	0.8309	0.5187	
Quadratic	0.0065	0.4234	0.9539	0.8322	Suggested
Cubic	0.4234		0.9572		Aliased

**Table 4:** The enzyme inhibition results of the samples.

Samples	Collagenase	Elastase	Hyaluronidase
	IC_50_ (µg/mL)		
TO-M	66.863 ± 1.653 ^b^	145.030 ± 8.681 ^c^	69.395 ± 9.065 ^b^
TO-O	35.166 ± 7.713 ^a^	96.846 ± 5.736 ^b^	64.936 ± 0.442 ^b^
RA	51.422 ± 0.979 ^a^	28.477 ± 1.27 ^a^	47.473 ± 1.053 ^a^
PC	45.027 ± 5.310 ^a^	20.462 ± 0.197 ^a^	63.996 ± 1.957 ^b^

TO-O: Rosmarinic acid-enriched extract of *T. orientalis*; TO-M: maceration extract of *T. orientalis*; RA: rosmarinic acid; PC: catechin for collagenase inhibition; epigallocatechin gallate (EGCG) for elastase inhibition; Tannic acid for hyaluronidase inhibition. Values bearing different lowercase letters within the same column are significantly different according to Tukey’s multiple-comparison test (*p* < 0.005), whereas values bearing the same letter are not significantly different.

**Table 5:** % wound closure values of samples at 6, 12 and 18 h.

Samples	6 h	12 h	18 h
Control	9.091 ± 3.134 ^c^	34.711 ± 2.574 ^b^	61.364 ± 3.102 ^a^
TO-M	17.320 ± 2.316 ^b^	34.845 ± 3.752 ^b^	51.753 ± 2.039 ^b^
TO-O	25.902 ± 1.885 ^a^	44.798 ± 2.251 ^a^	63.907 ± 1.992 ^a^
RA	11.951 ± 2.395 ^c^	16.098 ± 2.357 ^c^	41.220 ± 2.729 ^c^

Values bearing different lowercase letters within the same column are significantly different according to Tukey’s multiple-comparison test (*p* < 0.0001), whereas values bearing the same letter are not significantly different. TO-O: rosmarinic acid-enriched extract of *T. orientalis*; TO-M: maceration extract of *T. orientalis*; RA: rosmarinic acid; Control: cells maintained in culture medium without exposure to any test sample.

**Table 6:** The top 10 high-degree genes in the network.

Rank	Gene	Degree	Betweenness Centrality	Closeness Centrality
1	*TNF*	44	0.2155	0.6545
2	*IL1B*	41	0.1403	0.6316
3	*IL10*	30	0.0445	0.5714
4	*CCL2*	30	0.0608	0.5669
5	*EGFR*	27	0.0613	0.5581
6	*INS*	25	0.0841	0.5333
7	*PPARG*	22	0.0448	0.5180
8	*IL4*	21	0.0999	0.5217
9	*PTGS2*	21	0.0182	0.5255
10	*STAT1*	21	0.0321	0.5255

**Table 7:** The maximum and minimum values of the parameters determined in the experimental design.

Independent Variables	Minimum	Maximum
Extraction time (A)	60 s	120 s
Extraction temperature (B)	25 °C	50 °C
Solvent volume (C)	5 mL	10 mL
Microwave power (D)	300 W	500 W
Ultrasonic duration (E)	20 s	60 s
Vortex duration (F)	20 s	60 s

**Table 8:** Fractional factorial design matrix.

Line	A	B	C	D	E	F
1	60	50	10	500	20	60
2	120	25	5	300	20	60
3	120	50	10	500	20	20
4	60	25	5	300	20	20
5	60	50	10	300	60	60
6	60	25	10	500	20	20
7	60	25	10	300	20	60
8	60	50	10	300	20	20
9	60	25	5	500	60	20
10	120	50	5	500	20	60
11	120	50	5	300	60	60
12	120	25	10	300	20	20
13	60	25	5	500	20	60
14	120	25	10	500	20	60
15	120	50	10	500	60	60
16	60	25	5	300	60	60
17	120	50	10	300	20	60
18	120	25	10	300	60	60
19	60	25	10	500	60	60
20	60	50	5	300	20	60
21	60	50	5	300	60	20
22	60	50	5	500	20	20
23	60	25	10	300	60	20
24	120	50	10	300	60	20
25	120	50	5	300	20	20
26	60	50	5	500	60	60
27	120	25	5	500	20	20
28	120	25	5	500	60	60
29	120	25	5	300	60	20
30	60	50	10	500	60	20
31	120	50	5	500	60	20
32	120	25	10	500	60	20

**Table 9:** Box–Behnken experimental design matrix.

Line	A	B	C
1	90	50	10
2	90	25	20
3	90	37.5	15
4	60	50	15
5	60	37.5	20
6	120	25	15
7	60	37.5	10
8	90	50	20
9	120	50	15
10	90	37.5	15
11	60	25	15
12	90	37.5	15
13	120	37.5	20
14	90	25	10
15	90	37.5	15
16	120	37.5	10
17	90	37.5	15

## Data Availability

The original contributions presented in this study are included in the article/Supplementary Materials. Further inquiries can be directed to the corresponding author.

## Supplementary Materials

The following supporting information can be downloaded at https://www.mdpi.com/article/10.3390/ph19101633/s1, Supplementary File S1 (Table S1): Summary of the analytical validation parameters of the RP-HPLC method; Supplementary File S2 (Table S2): SwissADME-predicted physicochemical properties of rosmarinic acid; (Table S3): ProTox-3.0-predicted organ toxicity classification and probabilities of rosmarinic acid; (Figure S1): Toxicity prediction radar chart of rosmarinic acid; Supplementary File S3: Wound healing-related genes; Supplementary File S4: GO Biological Process (BP) Enrichment Analysis; Supplementary File S5: GO Cellular Component (CC) Enrichment Analysis; Supplementary File S6: GO Molecular Function (MF) Enrichment Analysis; Supplementary File S7: KEGG Pathway Enrichment Analysis.

## Abbreviations

The following abbreviations are used in this manuscript:

TGF-*β*	Transforming growth factor-beta
FGF	Fibroblast growth factor
PDGF	Platelet-derived growth factor
EGF	Epidermal growth factor
ECM	Extracellular matrix
MMP	Matrix metalloproteinase
RSM	Response surface methodology
HPLC	High-performance liquid chromatography
NF-*κ*B	Nuclear Factor Kappa B
IL-6	Interleukin-6
MMP-1	Matrix metalloproteinase-1
L929	Mouse fibroblast cells
TO-M	Maceration extract of *T. orientalis*
TO-O	Rosmarinic acid-enriched extract of *T. orientalis*
RA	Rosmarinic acid
EGCG	Epigallocatechin gallate
PC	Positive control
LOD	Limit of detection
LOQ	Limit of quantification
ADME	Absorption, distribution, metabolism, and excretion
TPSA	Topological polar surface area
BBB	Blood–brain barrier
IC_50_	Half-maximal inhibitory concentration
PI3K/AKT	Phosphatidylinositol 3-kinase/protein kinase B
AGE-RAGE	Advanced glycation end products–receptor for advanced glycation end products
