# Supplementary material for: The Network Pharmacology of Rosmarinic Acid and Elucidating the Wound Healing Potential of a Rosmarinic Acid-Enriched Trachystemon orientalis Extract
Source: Pharmaceuticals (Basel). 2026 Oct 15;19(10):1633. doi: 10.3390/ph19101633 (PMC13708215; doi:10.3390/ph19101633)
Supplement: Supplementary file 1 [file pharmaceuticals-19-01633-s001.zip › Supplementary File S1.pdf]

Table S1. Summary of the analytical validation parameters of the RP-HPLC method

Compound Name	R^2	Regression equation	Linear range (µg/mL)	LOD	LOQ	Precision			A%RSD	A%RSD	Resolution
						80	100	120	Inter-Day	Intra-Day	
Gallic acid	0.9999	y = 18409x + 28234	5-100	0.505	1.530	101.0 ± 0.25	100.0 ± 0.55	100.5.5 ± 0.52	0.897	0.839	1.708
Catechin	0.9997	y = 3128,2x + 2838,4	5-100	0.922	2.793	101.7 ± 0.27	102.2 ± 1.21	102.1 ± 2.05	0.557	0.714	3.536
p-OH benzoic acid	0.9995	y = 12651x + 20544	5-100	0.129	0.390	99.2 ± 1.24	100.9 ± 1.04	100.6.2 ± 1.75	0.248	0.100	6.498
Vanillic acid	0.9997	y = 12083x + 13845	5-100	0.079	0.238	100.3 ± 1.95	100.1.0 ± 1.95	100.5.0 ± 0.21	0.221	0.101	2.63
Ferulic acid	0.9996	y = 35315x + 43778	5-100	0.251	0.760	100.2 ± 1.03	99.5 ± 1.04	98.2 ± 1.04	0.411	0.168	10.773
p-Coumaric acid	0.9999	y = 12876x + 14959	5-100	0.934	2.831	100.1 ± 1.14	100.1 ± 1.07	100.2 ± 0.09	1.209	1.731	1.78
Sinapic acid	0.9998	y = 21937x + 2430,9	5-100	0.210	0.635	102.3 ± 1.97	98.9 ± 1.97	98.3 ± 0.15	0.533	1.477	1.82
Rosmarinic acid	0.9998	y = 8016,1x + 5885,4	5-100	0.628	1.903	102.8 ± 0.21	98.8 ± 0.92	98.2 ± 0.92	0.276	0.155	2.655
Quercetin	0.9999	y = 7753,7x + 450,26	5-100	0.505	1.530	98.5 ± 0.28	101.9 ± 0.88	102.4.9 ± 0.68	0.337	0.411	2.666

Validation

Precision was evaluated as repeatability, intra-day precision, and inter-day precision. Repeatability was assessed by six replicate injections at the same concentration under identical conditions. Intra-day precision was evaluated by analyzing three concentration levels (80, 100, and 120%) in triplicate within the same day, whereas inter-day precision was determined by repeating the analysis on three consecutive days. Precision was expressed as relative standard deviation (%RSD).

Accuracy was evaluated by recovery studies using the standard addition method at three concentration levels (80%, 100%, and 120%). Each level was analyzed in triplicate, and the results were expressed as mean recovery (%) ± SD.

Linearity was evaluated by analyzing five calibration levels (5-500 μg/mL). Calibration curves were constructed by plotting peak area against analyte concentration, and linear regression analysis was performed. The regression equation and correlation coefficient (R^2) were calculated for each analyte.

The specificity of the method was demonstrated by the absence of interfering peaks at the retention times of the analytes. Adequate chromatographic separation was confirmed by resolution ($Rs > 1.5$).

LOD and LOQ were calculated using the equations $LOD = 3.3\sigma/S$ and $LOQ = 10\sigma/S$, respectively.
