# Supplementary material for: The Network Pharmacology of Rosmarinic Acid and Elucidating the Wound Healing Potential of a Rosmarinic Acid-Enriched Trachystemon orientalis Extract
Source: Pharmaceuticals (Basel). 2026 Oct 15;19(10):1633. doi: 10.3390/ph19101633 (PMC13708215; doi:10.3390/ph19101633)
Supplement: Supplementary file 1 [file pharmaceuticals-19-01633-s001.zip › Supplementary File S2.pdf]

Table S2. SwissADME-predicted physicochemical properties of rosmarinic acid

Canonical SMILES
C1=CC(=C(C=C1C[C@H](C(=O)O)OC(=O)/C=C/C2=CC(=C(C=C2)O)O)O)O

Physicochemical Properties

Formula	C18H16O8
MW	360.31
Heavy atoms	26
Aromatic heavy atoms	12
Fraction Csp3	0.11
Rotatable bonds	7
H-bond acceptors	8
H-bond donors	5
MR	91.40
TPSA	144.52

Lipophilicity

Model	Log P
iLOGP	1.48
XLOGP3	2.36
WLOGP	1.65
MLOGP	0.90

Model	Log P
Silicos-IT Log P	1.50
Consensus Log P	1.58

Water Solubility

ESOL Log S	−3.44
ESOL Solubility (mg/ml)	1.31e-01
ESOL Solubility (mol/l)	3.63e-04
ESOL Class	Soluble
Ali Log S	−5.04
Ali Solubility (mg/ml)	3.32e-03
Ali Solubility (mol/l)	9.22e-06
Ali Class	Moderately soluble
Silicos-IT LogSw	−2.17
Silicos-IT Solubility (mg/ml)	2.41e+00
Silicos-IT Solubility (mol/l)	6.70e-03
Silicos-IT Class	Soluble

Pharmacokinetics

**Parameter**	**Prediction**
GI absorption	Low
BBB permeant	No

Parameter	Prediction
P-gp substrate	No
CYP1A2 inhibitor	No
CYP2C19 inhibitor	No
CYP2C9 inhibitor	No
CYP2D6 inhibitor	No
CYP3A4 inhibitor	No
Log Kp (skin permeation)	−6.82 cm/s

Drug-likeness

Filter	Result
Lipinski violations	0
Ghose	Yes
Veber	No; 1 violation (TPSA >140)
Egan	No; 1 violation (TPSA >131.6)
Muegge	Yes
Bioavailability score	0.56

Medicinal Chemistry

Parameter	Value
PAINS alerts	1 (catechol_A)
Brenk alerts	2 (catechol, michael_acceptor_1)

Parameter	Value
Lead-likeness	No; 1 violation (MW >350)
Synthetic accessibility	3.38

Table S3 ProTox-3.0-predicted organ toxicity classification and probabilities for R

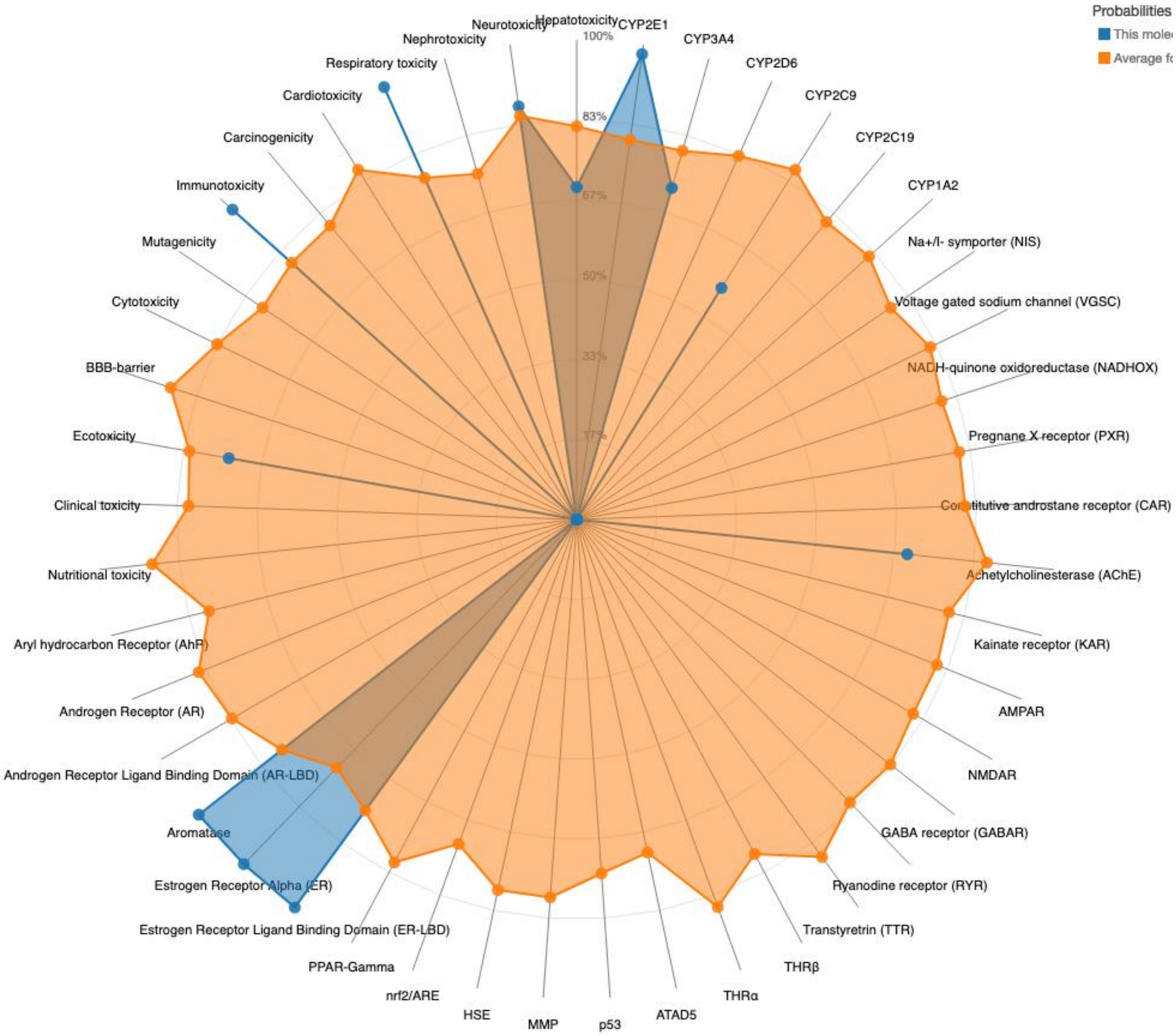

osmarinic acid.

Classification	Target	Shorthand	Prediction	Probability
Organ toxicity	Hepatotoxicity	dili	Inactive	0,62
Organ toxicity	Neurotoxicity	neuro	Inactive	0,88
Organ toxicity	Nephrotoxicity	nephro	Active	0,64
Organ toxicity	Respiratory toxicity	respi	Inactive	0,54
Organ toxicity	Cardiotoxicity	cardio	Inactive	0,69
Toxicity end points	Carcinogenicity	carcino	Inactive	0,66
Toxicity end points	Immunotoxicity	immuno	Active	0,93

Toxicity end points	Mutagenicity	mutagen	Inactive	0,85
Toxicity end points	Cytotoxicity	cyto	Inactive	0,90
Toxicity end points	BBB-barrier	bbb	Active	0,62
Toxicity end points	Ecotoxicity	eco	Inactive	0,79
Toxicity end points	Clinical toxicity	clinical	Active	0,59
Toxicity end points	Nutritional toxicity	nutri	Inactive	0,65
Tox21-Nuclear receptor signalling pathways	Aryl hydrocarbon Receptor (AhR)	nr_ahr	Inactive	0,82
Tox21-Nuclear receptor signalling pathways	Androgen Receptor (AR)	nr_ar	Inactive	0,94
Tox21-Nuclear receptor signalling pathways	Androgen Receptor Ligand Binding Domain (AR-LBD)	nr_ar_lbd	Inactive	0,99
Tox21-Nuclear receptor signalling pathways	Aromatase	nr_aromatase	Inactive	0,96
Tox21-Nuclear receptor signalling pathways	Estrogen Receptor Alpha (ER)	nr_er	Inactive	0,74
Tox21-Nuclear receptor signalling pathways	Estrogen Receptor Ligand Binding Domain (ER-LBD)	nr_er_lbd	Inactive	0,72
Tox21-Nuclear receptor signalling pathways	Peroxisome Proliferator Activated Receptor Gamma (PPAR-Gamma)	nr_ppar_gamma	Inactive	0,80
Tox21-Stress response pathways	Nuclear factor (erythroid-derived 2)-like 2/antioxidant responsive element (nrf2/ARE)	sr_are	Inactive	0,88

Tox21-Stress response pathways	Heat shock factor response element (HSE)	sr_hse	Inactive	0,88
Tox21-Stress response pathways	Mitochondrial Membrane Potential (MMP)	sr_mmp	Inactive	0,67
Tox21-Stress response pathways	Phosphoprotein (Tumor Supressor) p53	sr_p53	Inactive	0,73
Tox21-Stress response pathways	ATPase family AAA domain-containing protein 5 (ATAD5)	sr_atad5	Inactive	0,86
Molecular Initiating Events	Thyroid hormone receptor alpha (THRα)	mie_thr_alpha	Inactive	0,90
Molecular Initiating Events	Thyroid hormone receptor beta (THRβ)	mie_thr_beta	Inactive	0,78
Molecular Initiating Events	Transtyretrin (TTR)	mie_ttr	Inactive	0,97
Molecular Initiating Events	Ryanodine receptor (RYR)	mie_ryr	Inactive	0,98
Molecular Initiating Events	GABA receptor (GABAR)	mie_gabar	Inactive	0,96
Molecular Initiating Events	Glutamate N-methyl-D-aspartate receptor (NMDAR)	mie_nmdar	Inactive	0,92
Molecular Initiating Events	alpha-amino-3-hydroxy-5-methyl-4-isoxazolepropionate receptor (AMPAR)	mie_ampar	Inactive	0,97
Molecular Initiating Events	Kainate receptor (KAR)	mie_kar	Inactive	0,99
Molecular Initiating Events	Achetylcholinesterase (AChE)	mie_ache	Inactive	0,62
Molecular Initiating Events	Constitutive androstane receptor (CAR)	mie_car	Inactive	0,98
Molecular Initiating Events	Pregnane X receptor (PXR)	mie_pxr	Inactive	0,92

Molecular Initiating Events	NADH-quinone oxidoreductase (NADHOX)	mie_nadhox	Inactive	0,97
Molecular Initiating Events	Voltage gated sodium channel (VGSC)	mie_vgsc	Inactive	0,95
Molecular Initiating Events	Na+/I- symporter (NIS)	mie_nis	Inactive	0,98
Metabolism	Cytochrome CYP1A2	CYP1A2	Inactive	0,81
Metabolism	Cytochrome CYP2C19	CYP2C19	Inactive	0,92
Metabolism	Cytochrome CYP2C9	CYP2C9	Active	0,62
Metabolism	Cytochrome CYP2D6	CYP2D6	Inactive	0,81
Metabolism	Cytochrome CYP3A4	CYP3A4	Inactive	0,94
Metabolism	Cytochrome CYP2E1	CYP2E1	Inactive	1

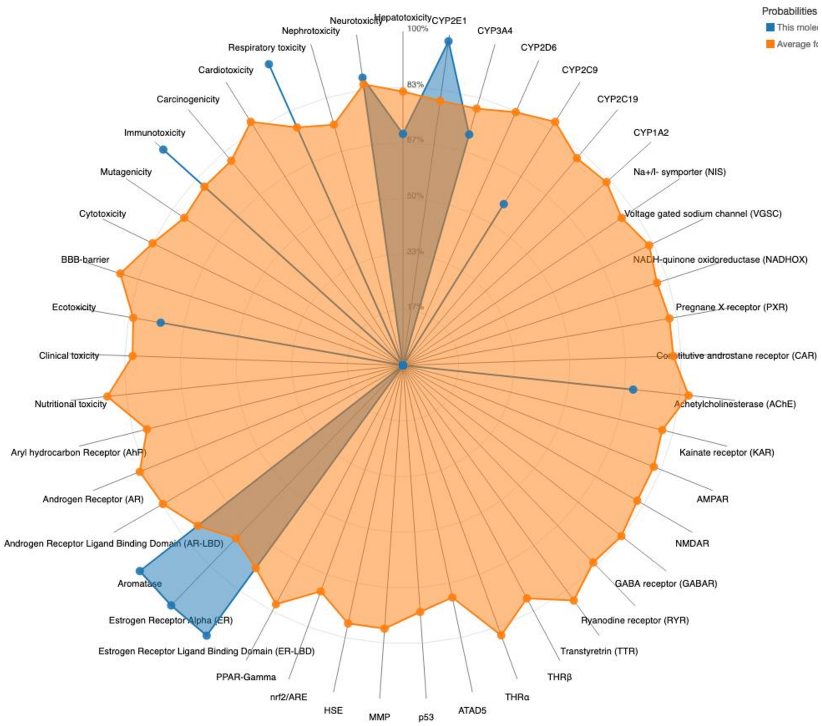

Figure S1 Toxicity Prediction Radar Chart of Rosmarinic acid.
